# Transcriptomic and proteomic insights into innate immunity and adaptations to a symbiotic lifestyle in the gutless marine worm *Olavius algarvensis*

**DOI:** 10.1186/s12864-016-3293-y

**Published:** 2016-11-21

**Authors:** Juliane Wippler, Manuel Kleiner, Christian Lott, Alexander Gruhl, Paul E. Abraham, Richard J. Giannone, Jacque C. Young, Robert L. Hettich, Nicole Dubilier

**Affiliations:** 1 Symbiosis Department, Max Planck Institute for Marine Microbiology, Celsiusstr. 1, D-28359 Bremen, Germany; 2 Energy Bioengineering and Geomicrobiology Research Group, University of Calgary, Calgary, T2N 1N4 AB Canada; 3 HYDRA Institute for Marine Sciences, Elba Field Station, Via del Forno 80, 57034 Campo nell’ Elba, (LI) Italy; 4 Oak Ridge National Laboratory, Chemical Sciences Division, Oak Ridge, Tennessee, 1 Bethel Valley Rd, Oak Ridge, TN 37831 USA; 5 Present Address: Saul Ewing LLP, 1500 Market Street, 37th Floor, Philadelphia, PA 19102-2186 USA; 6 Symbiosis Department, Max Planck Institute for Marine Microbiology, Celsiusstr. 1, D-28359 Bremen, Germany

**Keywords:** RNA-Seq, Annelida, Oligochaeta, Phallodrilinae, PGRP, FREP, SRCR, Respiratory pigment, Carbon monoxide, Immunology, Chemosynthetic symbiosis

## Abstract

**Background:**

The gutless marine worm *Olavius algarvensis* has a completely reduced digestive and excretory system, and lives in an obligate nutritional symbiosis with bacterial symbionts. While considerable knowledge has been gained of the symbionts, the host has remained largely unstudied. Here, we generated transcriptomes and proteomes of *O. algarvensis* to better understand how this annelid worm gains nutrition from its symbionts, how it adapted physiologically to a symbiotic lifestyle, and how its innate immune system recognizes and responds to its symbiotic microbiota.

**Results:**

Key adaptations to the symbiosis include (i) the expression of gut-specific digestive enzymes despite the absence of a gut, most likely for the digestion of symbionts in the host's epidermal cells; (ii) a modified hemoglobin that may bind hydrogen sulfide produced by two of the worm’s symbionts; and (iii) the expression of a very abundant protein for oxygen storage, hemerythrin, that could provide oxygen to the symbionts and the host under anoxic conditions. Additionally, we identified a large repertoire of proteins involved in interactions between the worm's innate immune system and its symbiotic microbiota, such as peptidoglycan recognition proteins, lectins, fibrinogen-related proteins, Toll and scavenger receptors, and antimicrobial proteins.

**Conclusions:**

We show how this worm, over the course of evolutionary time, has modified widely-used proteins and changed their expression patterns in adaptation to its symbiotic lifestyle and describe expressed components of the innate immune system in a marine oligochaete. Our results provide further support for the recent realization that animals have evolved within the context of their associations with microbes and that their adaptive responses to symbiotic microbiota have led to biological innovations.

**Electronic supplementary material:**

The online version of this article (doi:10.1186/s12864-016-3293-y) contains supplementary material, which is available to authorized users.

## Background

Most, if not all, animals are associated with a species-specific microbial assemblage that profoundly affects their evolution, ecology, development and health [1–3]. Animals and their microbiota have evolved molecular mechanisms to recognize and maintain these stable associations, and on the host side, these mechanisms are largely mediated by their immune system [4]. The mechanisms that govern host-symbiont interactions have been studied in a number of model organisms [4, 5], but remain unexplored in many animal phyla.

**Fig. 1:**
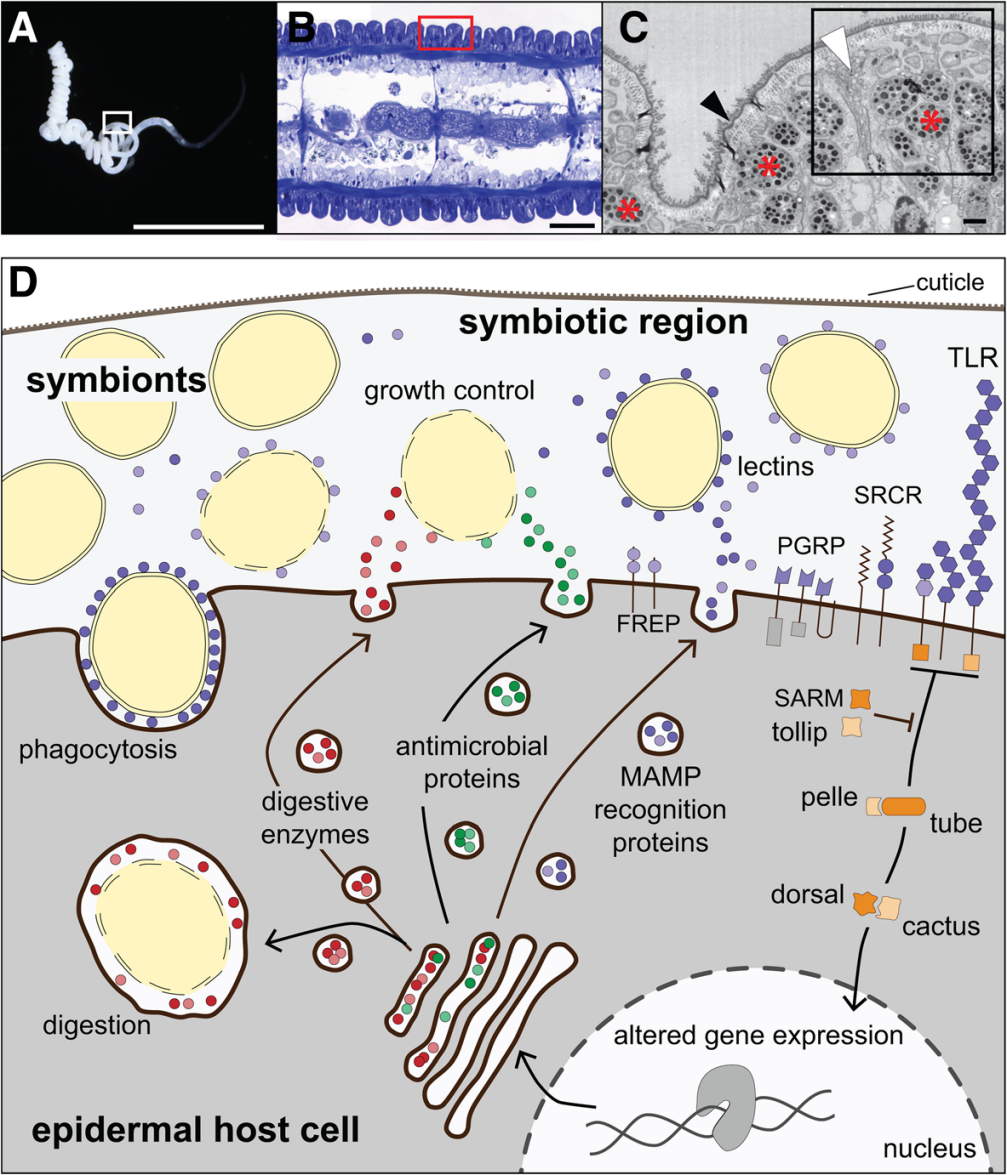
Schematic overview of proposed molecular host-symbiont interactions. **a**) Light micrograph of an *Olavius algarvensis* worm. Scale bar 5 mm. **b**) Light micrograph of a longitudinal section through *O. algarvensis*, tissue stained with toluidine blue. Scale bar 50 μm. C) Transmission electron micrograph of the symbiotic region, longitudinal section. Red asterisks, symbiont cells; black arrow, cuticle; white arrow, epidermal cell extensions. Scale bar 5 μm. The boxes in **a**), **b**) and **c**) frame regions corresponding to the image to their right (in **a**) and **b**)) or below (in **c**). Images **a**, **b**, and **c** do not show the same worm specimen. **d**) Schematic overview of the main groups of expressed pattern recognition molecules, components of the Toll immune signaling pathway and proposed interactions between the host and its symbionts. Ig, immunoglobulin domain proteins; PGRP, peptidoglycan recognition proteins; SRCR scavenger receptor-like cysteine rich proteins; TLR, Toll-like receptors; FREP, fibrinogen-related proteins; AMPs, antimicrobial proteins

*Olavius algarvensis* is a gutless oligochaete worm (Annelida; Oligochaeta; Phallodrilinae) that lives in an obligate nutritional symbiosis with at least four bacterial species [6]. These extracellular endosymbionts thrive in a dense bacterial layer between the cuticle and the epidermis of the worm (Fig. 1). Over the course of their symbiotic evolution, the gutless oligochaetes have lost their digestive and excretory organs, and rely solely on their bacterial symbionts for nourishment and removal of their waste products [7–9]. *O. algarvensis* harbors two gammaproteobacterial symbiont species that are chemoautotrophic sulfur oxidizers, and two deltaproteobacterial symbionts that are sulfate-reducing bacteria [6]. Together, these symbionts engage in a syntrophic sulfur cycle that enables autotrophic carbon fixation by the sulfur-oxidizing symbionts and provision of organic carbon to the host [8, 9]. Many worm individuals also harbor a spirochaetal symbiont, whose function has not yet been resolved [10].

Metagenomic and metaproteomic studies of the symbionts have revealed much about their metabolic capabilities, highlighted their immense capacity to use and recycle the host’s waste products and led to the discovery of novel, energy-efficient pathways to fix both inorganic and organic carbon into biomass [8, 9]. Research aimed at a better understanding of the host, on the other hand, has been hampered by the fact that the worms are very small (0.1–0.2 mm in diameter and 10–20 mm in length), cannot be cultivated, and by a lack of sequence data. Recent advances in sequencing technology have made it possible to sequence and assemble comprehensive *de novo* transcriptomes of uncultured, non-model organisms collected in the environment. These transcriptomes provide a reference database for identifying the proteins organisms express using mass-spectrometry-based proteomic approaches. This methodological advance has opened the door for in depth studies of the molecular repertoire used by *O. algarvensis* and other non-cultivable organisms to establish and maintain a successful symbiosis.

All animals employ mechanisms for selecting and maintaining a specific microbial consortium over the course of their lives, while avoiding overgrowth by their own microbiota or infection by detrimental bacteria from the environment. The innate immune system is crucial in the establishment and maintenance of healthy symbiotic interactions, but has so far not been studied in gutless oligochaetes. These hosts face additional challenges because they obligately rely on their symbionts and therefore must provide conditions under which they can thrive, while also dealing with the physiological challenges caused by their symbiotic lifestyle. For example, *O. algarvensis* must be able to live in anoxic sediment layers for extended periods of time to enable sulfate reduction by its anaerobic sulfate-reducing symbionts [8]. Additionally, it must be able to deal with the hydrogen sulfide that is produced during sulfate reduction. It must also be able to endure the relatively high carbon monoxide concentrations in its environment, which both the sulfate-reducing and sulfur-oxidizing symbionts use as an energy source [9, 11]. Another challenge occurs when *O. algarvensis* inhabits the upper oxygenated sediment layers where it competes for oxygen with its aerobic sulfur-oxidizing symbionts.

Here, we used transcriptomics and proteomics to elucidate how *O. algarvensis* fulfills the physiological requirements outlined above and how it obtains nutrition from its symbionts. We exposed worms collected from the environment to two types of conditions that they naturally encounter to increase transcriptome and proteome coverage. Our identification and analysis of proteins expressed by *O. algarvensis* provide insights into their molecular mechanisms for microbe recognition, interaction and regulation, as well as their physiological adaptations to living in symbiosis with sulfur-oxidizing and sulfate-reducing bacteria.

## Methods

### Sample collection and incubations

For proteomic analyses, sediment that contained gutless oligochaete worms was collected at 7 m water depth in the Bay of Sant’Andrea, Elba, Italy (42° 48’ 29.38’ N, 10° 08’ 31.57’ E) in October 2007 and 2008. Worms were carefully washed out of the sediment at the HYDRA field station (Fetovaia, Elba, Italy) by hand (for details see [9]). To increase proteome coverage we treated the worms in the following manner. Worms were either immediately frozen in liquid nitrogen in batches of 150–200 worms (called "fresh" worms in the following) or were kept for 8 days in glass petri dishes filled with a thin layer (2–3 mm) of washed sediment and 0.2 μm-filtered sea water and then frozen in liquid nitrogen (called "starved" worms in the following, because no external electron donor for energy conservation and autotrophic carbon fixation was provided). The sulfur-oxidizing symbionts of *O. algarvensis* store large amounts of sulfur and polyhydroxyalkanoate granules, which give the worms a bright white appearance. Under prolonged exposure to oxygen without access to an electron donor like the sulfide produced anaerobically by the sulfate-reducing symbionts, these storage granules become depleted, the worms turn transparent, and are effectively starved of nutrition. Transparent worms are regularly found in the environment, especially during the reproductive season of the worms. All samples were stored in liquid N_2_ and later at −80 °C until further use.

For transcriptomics, 100–120 worms were collected in April 2012 from the same site as for proteomics. The live worms were kept in washed sediment and transported to the lab in Bremen where they were washed out of the sediment again, washed in petri dishes with filtrated seawater, then flash-frozen in liquid nitrogen and stored at −80 °C until they were used to prepare the cDNA library “A”. A second collection of worms was used for library "B" to identify genes expressed under prolonged anoxia, a condition that the worms often experience. For library "B", 100–120 live worms were collected in March 2013 from the same site as above, transported to Bremen in the same way as for library "A", and incubated in anoxic serum bottles for 43 h. Serum bottles were filled with sediment and sea water from Elba, and were flushed with nitrogen to remove oxygen from the headspace. The sediment and sea water were not sterilized, so that fully anoxic conditions could develop quickly through microbial metabolism. Oxygen concentrations were measured at the end of the incubation with an oxygen microelectrode and were below 0.1 μM. Worms were fixed overnight in RNAlater (Thermo Fisher Scientific, Braunschweig, Germany) at 4 °C and stored at −80 °C until they were used to prepare the cDNA library “B”.

### Illumina library preparation and sequencing

Total RNA was isolated using peqGOLD TriFast reagent (PEQLAB, Erlangen, Germany) and treated with DNase. Poly(A) + RNA was isolated from the total RNA, fragmented with ultrasound (2 pulses of 30 s at 4 °C) and used for cDNA synthesis with random hexamer primers. Illumina TruSeq adaptors were ligated to the ends of the cDNA fragments and amplified according to the manufacturer’s instructions (Illumina Inc., USA). Library DNA fragments of 300–500 bp were eluted from a preparative agarose gel and paired-end sequenced on an Illumina HiSeq2000 sequencer (2x 100 bp). A standard PhiX174 DNA spike-in of 1% was used for sequencing quality control. We sequenced ~170 million read pairs from library A, and ~6 million read pairs from library B (Additional file 1: Table S1). For library B, a much smaller number of reads was sequenced because the purpose of this library was to detect abundant transcripts expressed under anoxic conditions.

### *De novo* transcriptome assembly and sequence analysis

The raw reads were trimmed to remove Illumina adapters, filtered for PhiX174 spike-in DNA and quality trimmed with nesoni clip version 0.109 (parameters used: −-match 7 –quality 27 –trim-start 10) [12]. The cleaned reads from library A and B were co-assembled *de novo* using Trinity release 2013-02-25 with default parameters [13]. Trinity reports individual assembled transcript sequences (“isoforms”) as members of transcript families (“components”), which can represent fragments of the same transcript, chimeric artifacts, or actual biological splice variants of a gene. In our reports of numbers of different transcripts from a certain family of proteins (e.g. number of identified peptidoglycan recognition proteins), we stick to the more conservative number of “components” rather than reported isoforms, as these are difficult to reliably verify without a confirmed reference. Transcripts were quantified with RSEM as implemented in Trinity using default parameters [14].

*De novo* assembled transcripts were annotated with blast2GO [15]. Transcripts of particular interest were searched against the invertebrate division of EST (Expressed Sequence Tags) and TSA (Transcriptome Shotgun Assembly) sequences of NCBI with tblastx [16] to determine their similarity to genes expressed in other annelids.

Hemoglobin sequences were assigned to families, if possible, based on sequence homology and specific conserved amino acid patterns as described in [17]. The secondary structures of the putative sulfide binding domains in *O. algarvensis* hemoglobin chains were predicted with hydrophobic cluster analysis using the program drawhca [18].

### Host species identification

Two species of gutless oligochaete co-occur at the sampling site, which can only be distinguished under the dissecting scope when sexually mature. The majority of worms used in this study were not sexually mature, and as a result, could not be morphologically identified. Therefore, we used EMIRGE [19] to estimate the relative abundance of the different species in our samples based on the read coverage of the mitochondrial cytochrome c oxidase I (COI) gene. We determined that the contamination with species other than *Olavius algarvensis* was less than 3.5% in library A and less than 0.1% in library B.

### 2D-LC-MS/MS

Protein was extracted from frozen worms, and peptides prepared as previously described using a single-tube small processing method [9, 20]. We analyzed three biological replicates for each condition (fresh and starved). All samples were analyzed in technical duplicates via 24 h nano-2D-LC MS/MS with a split-phase column (RP-SCX-RP) [21, 22] on a hybrid linear ion trap-Orbitrap (Thermo Fischer Scientific), as previously described [9].

### Peptide and protein identifications

Coding sequences (CDS) were predicted from the transcriptomes using FrameDP [23] and getorf of the EMBOSS package using the standard genetic code [24]. Transcriptome CDS were combined into a single protein sequence database with the symbiont protein sequence database used by Kleiner et al. 2012 [9]. To remove potential chimeric sequences and redundant CDS from the database we used sequence clustering with CD-HIT (version 4.5.4, [25]). Experimental peptide fragmentation spectra (MS/MS) generated from Xcalibur v.2.0.7 were compared with theoretical peptide fragmentation spectra obtained from the protein sequence database to which protein sequences of common contaminant proteins (e.g., human keratin and trypsin) were added to a total of 1,318,114 entries. To determine the false-discovery rate (FDR), a decoy database, generated by reversing the sequences of the target database, was appended.

MyriMatch v2.1.111 [26] was configured to derive fully-tryptic peptides with the following parameters: 2 missed cleavages, parent mass tolerance of 10 ppm, and a fragment mass tolerance of 0.5 m/z units. For protein inference, peptide identifications were merged together in IDPicker v.3 [27]. Only protein identifications with at least two identified spectra and a maximum q-value of 0.02 were considered for further analysis. The number of distinct peptides (i.e., a peptide with a unique series of amino acids, but does not relate to its uniqueness to the protein reference database) required for identifications was set to 1 to allow for the identification of small antimicrobial proteins and/or small, fragmented protein sequences in the transcriptome assembly. Based on these settings, protein-level FDR was < 3% for all samples.

To deal with sequence redundancy, post-search protein grouping was performed by clustering all protein sequences in the protein sequence database by sequence similarity (≥90%) using the UCLUST component of the USEARCH v5.0 software platform [28]. As described previously [29], identified proteins were then consolidated into their defined protein groups. Protein groups were represented by the longest protein sequence (i.e., the seed sequence), which shares ≥ 90% sequence similarity to each member of the protein group. Peptide uniqueness was re-assessed and classified as either unique (i.e., only belonging to one protein group) or non-unique (i.e., shared among multiple protein groups). We required each protein group to have at least two distinct peptides, with at least one of these being a unique peptide. For shared peptides belonging to multiple protein groups, their spectral counts were recalculated based on the proportion of uniquely identified peptides between the protein groups sharing the peptide. Following spectra balancing, total spectral counts of a protein group were converted to normalized spectra counts (nSpC) [30], which are derived from normalized spectral abundance factors [31]. Relative protein abundances of host proteins are listed in tables as nSpC values multiplied by 10,000 i.e. the sum of all host protein nSpC values in one sample is 10,000 and the nSpC values are thus given as a fraction of 10,000 (^0^/_000_).

## Results and Discussion

### Transcriptome/proteome measurement metrics

To generate our protein sequence database for host protein identification, we sequenced the transcriptomes of untreated whole worms (library A), and of worms kept under anoxic conditions for 43 h (library B). We chose these two conditions, which the worms encounter regularly, to obtain a larger range of host transcripts for the generation of a comprehensive reference sequence database for improved protein identification. After trimming and error correction, 159,551,509 (library A) and 5,745,537 (library B) read pairs remained, which were co-assembled into 173,602 contigs, with an N50 of 1236 bp, and 23,719 contigs of at least 1 kbp length (for more details on sequencing and assembly metrics, see Additional files 1: Table S1 and Table S2). Of these contigs, 31,913 could be functionally annotated (see Additional file 1: Figure S1 for annotation summary). The sequencing depth and assembly metrics are comparable to other, well-covered, transcriptomes of recently sequenced annelid taxa [32]. We analyzed proteomes of freshly collected worms, and worms that had been starved for 8 days - that is kept under oxic conditions without an external electron donor for energy conservation and autotrophic carbon fixation (see Methods). The purpose of creating these two conditions was to identify as many proteins as possible, including those expressed in worms that are starved. We identified a total of 4355 protein groups (see Methods for details on protein grouping) with a per sample protein-level FDR <3%. Of these, 2562 were host proteins and 1793 were symbiont proteins*.* The annotated host transcriptomes and proteomes were manually screened for sequences relevant for host-symbiont interactions. We identified 316 transcriptome sequences and 60 proteins potentially involved in microbe recognition, microbial growth regulation, symbiont digestion, immune modulation and physiological interactions (see Table 1 and Fig. 1).

**Table 1:** Overview of proposed host-symbiont interaction proteins

Functional category	Protein family	Transcripts ^a)^	Proteins ^b)^
*Pattern recognition proteins*			
	peptidoglycan recognition proteins (PGRPs)	6 (16)	1
	C-type lectins	33 (119)	5
	R-type lectins	6 (18)	4
	SUEL/rhamnose-binding lectins	7 (22)	1
	galectins	3 (3)	1
	fucolectin	1 (23)	0
	fibrinogen-related proteins (FREPs)	27 (161)	1
	toll-like/variable lymphocyte receptor-like (TLR/VLR)	13 (52)	1
	scavenger receptor cysteine-rich (SRCR) domain proteins	25 (164)	1
	beta-1,3-glucan binding protein	1 (1)	1
	novel immunoglobulin I-set proteins	16 (17)	1
	novel immunoglobulin V-set proteins	9 (22)	1
*Antimicrobial proteins*			
	lumbricin	1 (1)	1
	invertebrate-type lysozyme	1 (1)	0
	bactericidal permeability increasing protein BPI	1 (3)	1
	insect defensin/reeler-like proteins	4 (8)	1
	cysteine-rich secretory proteins (CRSPs)	6 (28)	2
	membrane attack complex/perforin	2 (23)	0
*Other immune effectors*			
	ROS modulator 1	2 (3)	1
	alpha-2-macroglobulin	10 (24)	1
	kazal-type serpin	2 (8)	0
	kunitz-type serpin	1 (2)	0
	leukocyte elastase inhibitors	5 (25)	1
	phosphatidylethanolamine-binding protein PEBP	3 (5)	1
*Immune response regulators*			
	Toll/interleukin-1 receptor (TIR) domain proteins	5 (9)	0
	NF-kappa-B inhibitor Cactus	2 (6)	0
	dorsal protein	2 (3)	1
	evolutionarily conserved signaling intermediate in Toll (ECSIT)	1 (1)	0
	Pelle protein	1 (1)	0
	Relish protein	1 (6)	0
	mitogen-activated protein kinase kinase kinase 7 (TAK1)	1 (1)	0
	I-kappa-B-kinase alpha (IKK α)	1 (1)	0
	I-kappa-B-kinase beta (IKK β)	1 (5)	0
	interleukin-1 receptor-associated kinase 1 (IRAK1)	1 (2)	0
	mitogen-activated protein kinase kinase kinase 4 (MEKK4)	1 (1)	0
	sterile alpha and TIR motif-containing protein (SARM)	1 (3)	0
	Toll-interacting protein Tollip	1 (2)	1
	(LPS-induced) tumor necrosis factor (TNF)	3 (7)	0
	Tumor necrosis factor (TNF)	4 (6)	0
	tumor necrosis factor alpha-induced protein 3 (TNFAIP3)	1 (3)	0
	tumor necrosis factor receptor associated proteins (TRAF)	9 (16)	0
	IFN regulatory factor	8 (10)	0
	IFN regulatory factor-binding protein	1 (1)	0
	IFN-induced GTPase	7 (23)	1
	macrophage migration inhibitory factor (MIF)	3 (24)	1
	ILN enhancer binding factor 2	2 (4)	1
	ILN-16	1 (1)	0
*Digestive enzymes*			
	carboxypeptidases	11 (22)	0
	cathepsins total	15 (28)	4
	cathepsin B	3 (12)	1
	cathepsin C	2 (4)	0
	cathepsin F	3 (3)	1
	cathepsin L	5 (6)	2
	cathepsin O	1 (2)	0
	cathepsin Z	1 (1)	0
	chymotrypsins	3 (17)	1
	pancreatic elastase	1 (1)	1
	alpha amylase	2 (3)	1
	lysosomal alpha glucosidase	1 (3)	1
	acid trehalase	2 (4)	1
	sucrase-isomaltase	1 (1)	0
	lysosomal acid lipase	1 (1)	0
*Respiration*			
	hemerythrin	2 (2)	2
	giant extracellular hemoglobin, globin chains	12 (16)	8
	giant extracellular hemoglobin, linker chains	6 (18)	5
sum		316 (1032)	60

For more extensive details see Additional file 2: Table S8

^a)^Number of transcripts defined as Trinity components, which approximately correspond to genes; see [13]; in parentheses: number of contigs (isoforms or fragments)

^b)^Number of unique proteins

### Physiological adaptations of the host to the symbiosis

#### Nutrients are transferred from the symbionts to the host via digestion

Previous to this study, it was not clear how gutless oligochaetes gain nutrition from their bacterial symbionts. Two transfer modes, which are not mutually exclusive, have been suggested for symbioses with endosymbionts [33]: (1) “milking” of the symbionts (uptake of small compounds leaked or actively released by the symbionts), and (2) symbiont digestion through endocytosis. Endocytosis can include phagocytosis of symbiont particles or whole cells, as well as uptake of extracellularly digested and dissolved compounds by pinocytosis.

Several results from this study indicate that the main mode of nutrient transfer from the symbionts to *O. algarvensis* is through their digestion. First, we measured significantly less symbiont protein relative to host protein in the proteomes of starved worms compared to freshly collected worms (*t*-test, *p* < 0.01). Symbiont protein accounted for only 18.7% of the total holobiont protein in starved worms, while freshly collected worms had 29.5% symbiont protein (Table 2 and Additional file 1: Table S3). In starved worms, the symbionts had no access to external sources of energy and carbon. Since these worms gain all their nutrition from their symbionts, the absence of external energy and carbon sources meant that no net growth of the symbiosis was possible. Thus, both the worms and their symbionts were starved of nutrition. We therefore hypothesize that the observed decrease in total symbiont protein relative to total host protein in starved worms occurred because the symbionts were digested by the host. An alternative explanation for the decrease in relative symbiont protein is that the symbionts, but not the host, degraded their own proteins in response to starvation.

**Table 2:** Difference in symbiont protein content in fresh worms compared to starved worms

	Symbionts fresh worms	Symbionts starved worms
average nSpC	3050.30	1869.77
standard deviation	421.88	122.47
# replicates	3	3
p-value *t*-test	0.00963	

Significant differences between fresh and starved samples were determined with the Student's *t*-Test; nSpC, normalized spectral counts. For more extensive details see Additional file 1: Table S3

Second, we identified 15 digestive enzymes predicted to occur in lysosomes, indicating their role in endocytosis, and 28 digestive enzymes involved in general secretory pathways, which could be targeted to phagolysosomes or to the extracellular region (Table 3). If these enzymes are not directed to phagolysosomes, but rather secreted extracellularly, they could also aid in the digestion of symbionts in the extracellular space just below the worm's cuticle, and precede endocytotic digestion by the epidermal cells. The digestive proteins included various proteases for the degradation of polypeptides and oligopeptides, glucosidases with specificity for α1 → 4, α1 → 6 and β1 → 4 glycosidic bonds, and enzymes involved in lipid and peptidoglycan degradation (Table 3).

**Table 3:** Digestive enzymes expressed in *Olavius algarvensis*

Transcript ID ^a)^	Protein ID	Annotation	Consensus localization evidence^b)^	Substrate or function
*Protein digestion*				
comp310626_c3	n.d.	pancreatic carboxypeptidase A1	secreted	release of C-terminal amino acids
comp330196_c1	n.d.	pancreatic carboxypeptidase A2	secreted	release of C-terminal amino acids
comp209868_c0	n.d.	pancreatic carboxypeptidase A2	secreted	release of C-terminal amino acids
comp329532_c3	n.d.	pancreatic carboxypeptidase A2	secreted or membrane	release of C-terminal amino acids
comp328734_c12	n.d.	carboxypeptidase	secreted	peptides
comp328734_c1	n.d.	carboxypeptidase	secreted	peptides
comp328734_c4	n.d.	carboxypeptidase	secreted	peptides
comp326419_c0	n.d.	uncharacterized carboxypeptidase	secreted	peptides
comp330196_c2	n.d.	uncharacterized carboxypeptidase	secreted	peptides
comp319717_c3	n.d.	lysosomal Pro-X carboxypeptidase	lysosomal	proline - amino acid bonds
comp320275_c1	n.d.	chymotrypsin A	secreted	Tyr-/Trp-/Phe-/Leu-|-Xaa bonds
comp306409_c1	n.d.	chymotrypsin B	secreted	Tyr-/Trp-/Phe-/Leu-|-Xaa bonds
comp334148_c2	BF11_334148_c2_seq1_11 BF11_334148_c2_seq2_10	chymotrypsin-like protease ctrl-1	secreted	proteins
comp331491_c1	BF11_331491_c1_seq1_7	pancreatic elastase	secreted	proteins
comp334775_c2	n.d.	cathepsin B	secreted	Arg – Arg/–Xaa bonds
comp335560_c0	BF11_335560_c0_seq5_5 BF11_335560_c0_seq9_5	cathepsin B	lysosomal	Arg – Arg/–Xaa bonds
comp306522_c2	n.d.	cathepsin F	lysosomal	peptides, cleaves Phe/Leu
comp306522_c3	n.d.	cathepsin F	lysosomal	peptides, cleaves Phe/Leu
comp308536_c0	BF11_308536_c0_seq1_12	cathepsin F	lysosomal	peptides, cleaves Phe/Leu
comp283346_c1	n.d.	cathepsin L	lysosomal	proteins
comp306922_c1	BF11_306922_c1_seq1_34	cathepsin L	lysosomal	proteins
comp315575_c0	n.d.	cathepsin L	lysosomal	proteins
comp315575_c2	FD_315575_c2_seq1:64:270:1:+	cathepsin L	lysosomal	proteins
comp328653_c0	n.d.	cathepsin L	lysosomal	proteins
comp329800_c6	n.d.	cathepsin O	lysosomal	peptides (endopeptidase)
comp314408_c1	n.d.	cathepsin Z	lysosomal	C-terminal amino acids (not Pro)
comp308700_c0	n.d.	cathepsin C (dipeptidyl peptidase I)	lysosomal	release of an N-terminal dipeptide
comp329456_c1	n.d.	cathepsin C (dipeptidyl peptidase I)	lysosomal	release of an N-terminal dipeptide
comp328746_c4	n.d.	alpha amylase	secreted	α1- > 4 glycosidic bonds
comp324906_c1	BF11_324906_c1_seq1_9 BF11_324906_c1_seq2_12	alpha amylase	secreted or membrane	α1- > 4 glycosidic bonds
comp335205_c1	BF11_335205_c1_seq1_20	lysosomal alpha glucosidase	secreted or membrane	α1- > 4 glycosidic bonds
comp320084_c0	n.d.	lysosomal beta-mannosidase	lysosomal	cleaves terminal β-D-mannose
comp334411_c3	n.d.	sucrase-isomaltase	secreted or membrane	α1- > 6 glycosidic bonds
comp329957_c8	BF11_329957_c8_seq2_17	acid trehalase	secreted or membrane	trehalose - > glucose
comp335402_c7	n.d.	acid trehalase	secreted or membrane	trehalose - > glucose
*Lipid degradation*				
comp22535_c0	n.d.	lysosomal acid lipase	secreted	hydrolyzes steryl esters
comp249291_c0	n.d.	lysozyme	secreted	peptidoglycan (glycosidic bonds)
comp250229_c0	BF11_250229_c0_seq1_15 BF11_250229_c0_seq2_17	peptidoglycan recognition protein	secreted	peptidoglycan (peptide bonds)
comp335695_c10	n.d.	peptidoglycan recognition protein	secreted or membrane	peptidoglycan (peptide bonds)
comp330541_c4	n.d.	peptidoglycan recognition protein	secreted	peptidoglycan (peptide bonds)
comp314994_c0	n.d.	peptidoglycan recognition protein	secreted or membrane	peptidoglycan (peptide bonds)
comp332570_c2	n.d.	peptidoglycan recognition protein	secreted or membrane	peptidoglycan (peptide bonds)

^a)^Defined as Trinity components; see [13]

^b)^Probable subcellular localization of proteins based on the results of TMHMM [117, 118], SignalP-4 [119], Phobius [120], TargetP [121], DISTILL [122], LocTree3 [123], BaCelLo [124], and iLoc-Animal [125]. *Secreted* – enters secretory pathway and is either excreted to the extracellular space or confined to non-cytoplasmic insides of intracellular compartments; *membrane* – predicted to be membrane integral; *lysosomal* – predicted to be targeted towards the lysosome. See Additional file 3: Table S14 for details on localization evidence

**Fig. 2:**
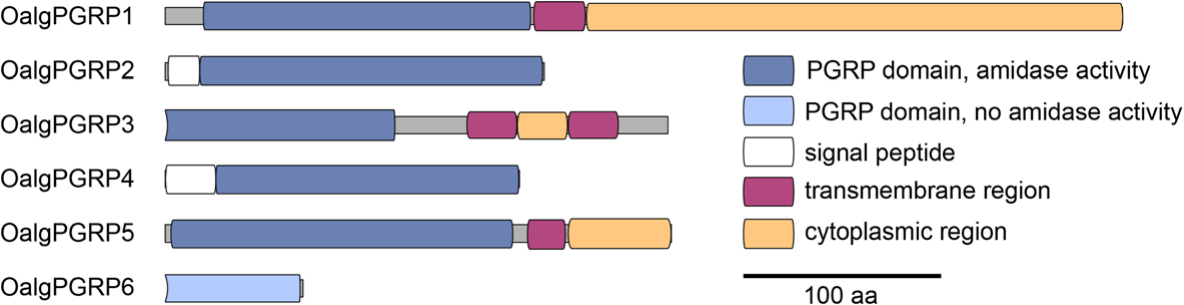
Domain structures of peptidoglycan recognition proteins. Structure of conserved functional domains in *Olavius algarvensis* peptidoglycan recognition proteins; OalgPGRP1: comp330541_c4; OalgPGRP2: comp250229_c0; OalgPGRP3: comp335695_c10; OalgPGRP4: comp314994_c0; OalgPGRP5: comp332570_c2; OalgPGRP6: comp1100768_c0

The third line of evidence that indicates that *O. algarvensis* digests its symbionts is that it expressed three different types of intestinal digestive enzymes, despite the fact that it does not have a mouth or gut. (i) The first type were digestive proteases (Table 3), namely pancreatic carboxypeptidase A, chymotrypsins A and B, cathepsins B, F and L, and pancreatic elastase. These enzymes are most often found in the intestinal tract of animals with a digestive system (Additional file 1: Table S4). Most of the *O. algarvensis* digestive proteases were highly similar to enzymes expressed in the midgut of the oligochaete *Eisenia andrei* (Additional file 1: Table S5). (ii) *O. algarvensis* also expressed a number of digestive glucosidases: two alpha amylases, with best BLAST hits to salivary gland and pancreatic amylases, an intestinal sucrase-isomaltase and two enzymes similar to pancreatic acid trehalase (Additional file 1: Table S6). (iii) *O. algarvensis* expressed five peptidoglycan recognition proteins (PGRPs) with predicted amidase activity (Fig. 2) and a lysozyme, all proteins that degrade peptidoglycans. Although PGRPs and lysozyme are known for their role in immune defense [34], they can also aid in the digestion of food bacteria [35, 36]. The five *O. algarvensis* PGRP sequences were highly similar to PGRPs expressed by the annelid *Eisenia andrei* in its midgut (Additional file 1: Table S5). The expression levels of these enzymes in starved worms were comparable to those in fresh worms, suggesting that there was no overall increase in host digestion rates of symbionts during starvation.

Taken together, these results strongly indicate that *O. algarvensis* obtains nutrition from its symbionts by digestion, rather than milking, using a wide range of digestive enzymes, many of which are known to be expressed in the digestive tissues of animals. Given that the symbiotic bacteria are only found in the body wall of their host, it is highly likely that, in adaptation to the symbiosis, the expression of these “intestinal” enzymes has been redirected from the gut to the epidermis. This assumption is supported by ultrastructural analyses that show the lysis of symbionts in the epidermal cells of the worm [37]. Additional support for the digestion of symbionts instead of “milking” stems from the observation that some of the *O. algarvensis* symbionts abundantly expressed high-affinity uptake transporters for organic substrates [9]. If 'milking' were the main manner in which the hosts gained their nutrition, they would compete with their symbionts for the uptake of small organic compounds.

#### Giant hemoglobins are likely involved in sulfide tolerance and transport

*O. algarvensis* abundantly expressed giant extracellular hemoglobins, which are respiratory pigments produced exclusively by annelids [38]. They are large multiprotein complexes (3.8 MDa in earthworms [39]), each consisting of more than a hundred copies of heme-containing globin chains and non-heme linker chains [38]. We found 12 globin chains and 6 linker chains from giant extracellular hemoglobins in our proteomes and transcriptomes (Additional file 1: Table S7). A signal peptide was predicted for all complete coding sequences, lending further support that these hemoglobins are indeed extracellular. Of the twelve *O. algarvensis* hemoglobin chain sequences, five could be unequivocally assigned to their respective families (3x family A, 2x family B).

**Fig. 3:**
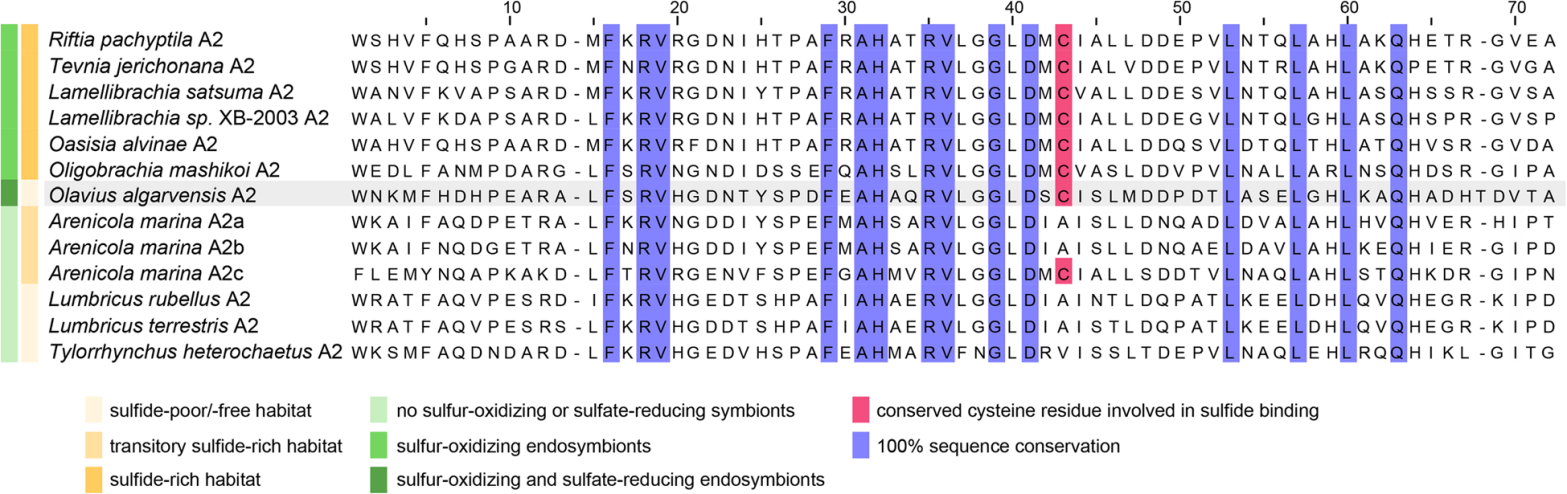
Protein alignment of hemoglobin A2 chains. Protein alignment of hemoglobin A2 chains from marine and terrestrial annelids: *Riftia pachyptila* (GenBank accession number: CAD29155), *Tevnia jerichonana* (GenBank accession number: AAP04530), *Lamellibrachia satsuma* (GenBank accession number: BAN58231), *Lamellibrachia sp*. XB-2003 (GenBank accession number: AAP04528), *Oasisia alvinae* (GenBank accession number: AAP04531), *Oligobrachia mashikoi* (GenBank accession number: Q7M413), *Arenicola marina* (GenBank accession numbers: A2a, CAI56308; A2b, CAJ32740; A2c, CAJ32741), *Lumbricus rubellus* (GenBank accession number: BF422675.2), *Lumbricus terrestris* (GenBank accession number: P02218), *Tylorrhynchus heterochaetus* (GenBank accession number: P09966) and *Olavius algarvensis* (comp287449_c0_seq1)

We found that one of the three chains assigned to family A contained a free cysteine residue (Fig. 3). Free cysteine residues do not participate in the formation of disulfide bonds in proteins, and therefore may unintentionally react with other blood components and disturb blood homeostasis [40, 41]. Extracellular hemoglobins are therefore under strong selective pressure to avoid the incorporation of free cysteines. The exception are annelids that experience high concentrations of sulfide in their habitats (Fig. 3, [17]). In these worms, free cysteine residues in the A2 and B2 hemoglobin chains may allow them to reversibly bind environmental hydrogen sulfide and oxygen simultaneously [42]. It has been argued that this could mitigate the toxic effects of hydrogen sulfide for these worms. In hydrothermal vent tube worms, which also have free cysteine residues in their hemoglobin, it is assumed that these also allow them to bind and transport sulfide to their sulfur-oxidizing endosymbionts [43]. In these worms, sulfide-binding to hemoglobin could also be mediated by zinc ions rather than free cysteine [44, 45]; however zinc does not appear to play a role in sulfide-binding in other annelids [46].

In *O. algarvensis*, the free cysteine residue is located in the conserved position that allows sulfide binding, and hydrophobic cluster analysis showed that the molecular environment of this free cysteine is highly similar to the sulfide-binding domain of A2 chains in other annelids (Additional file 1: Figure S2). It is therefore plausible that the *O. algarvensis* hemoglobin can also bind sulfide.

*O. algarvensis* lives in oligotrophic sediments with very low environmental sulfide concentrations [6, 9]. However, its sulfate-reducing symbionts are a considerable internal source of sulfide under anoxic conditions [6]. With its sulfide-binding hemoglobin, the host could store this internally produced sulfide for use by the SOX symbionts once they return to oxic conditions. Furthermore, the sulfide-binding hemoglobin might keep sulfide levels low in sensitive tissues of *O. algarvensis* such as the central nervous system.

#### Hemerythrin may enable respiration in the absence of O_2_ and in the presence of CO

In addition to hemoglobin, the host expressed two hemerythrins, which are also respiratory proteins, but without heme groups. One of these hemerythrins was by far the most abundant protein in both fresh and starved worms, and accounted for 11–15% of total host protein (Additional file 2: Table S8). In comparison, the second most abundant protein, a histone, accounted only for less than 3%. Both hemerythrins were more highly expressed than any of the hemoglobin chains; expression levels of the most abundant hemerythrin were almost 32 times higher than the most abundant globin chain in the proteome (Additional file 2: Table S8). Such abundant expression of hemerythrin is unknown from gut-bearing oligochaetes and other annelids (Additional file 1: Table S9).

Hemerythrin is an oxygen-carrying protein in sipunculids, priapulids and brachiopods, and also in a few polychaete annelids [47, 48]. In addition to oxygen transport, annelids might use hemerythrins for heavy metal resistance and antibacterial defense, or as an egg yolk protein [49–51]. In the only study that found hemerythrin expression in an oligochaete, it was assumed to be involved in heavy metal detoxification [50]. Since the environment of the *O. algarvensis* sampled for this study is not contaminated with high levels of heavy metals or pathogenic bacteria, and the worms in our experiments were not exposed to such conditions, it is unlikely that the high expression levels of hemerythrin are related to heavy metal resistance or antibacterial defense. We can also exclude its role in egg yolk protein, because the worms for proteomics were sampled in the fall, a time of the year when *O. algarvensis* does not reproduce (Kleiner, Lott, Wippler, unpublished observation). Therefore, it seems most likely that the hemerythrin in *O. algarvensis* is used to bind oxygen. This raises the question why *O. algarvensis* has two abundant oxygen binding proteins - hemoglobin and hemerythrin.

The fact that hemerythrin expression is unusual in oligochaetes suggests that there is a considerable selective advantage for its expression in *O. algarvensis*. One intriguing property of hemerythrin is that it is insensitive to carbon monoxide (CO) [52]. In contrast, heme proteins such as hemoglobin and myoglobin have much higher affinities for CO than for oxygen [53, 54]. This makes CO highly toxic to organisms that rely on heme proteins for oxygen transport. Considerable *in situ* CO concentrations of up to 51 nM were regularly measured in the *O. algarvensis* environment [11], and CO serves as an energy source for its sufur-oxidizing and sulfate-reducing symbionts [9]. Thus, the selective advantage of using hemerythrin for oxygen binding could be that it mitigates the adverse effects of carbon monoxide for the host.

The question remains why hemoglobin is also expressed in *O. algarvensis*, in parallel to hemerythrin. We speculate that hemerythrin and hemoglobin fulfill different functions in these worms. We propose that hemerythrin is used for oxygen storage to bridge the frequent and extended periods of anoxia that *O. algarvensis* is exposed to in the reduced sediment layers it mainly inhabits. Hemerythrin is well suited for oxygen storage because its oxygen binding capacity is stable under varying concentrations of O_2_, CO_2_ and protons [55, 56], and has been shown to play a key role in oxygen storage for bridging hypoxic episodes in sipunculids [57]. In contrast, hemoglobin, due to cooperative binding of oxygen and the Bohr effect, is well suited for gas exchange with the environment, which occurs in the upper oxic layer of the sediment where CO concentrations are much lower [11].

Interestingly, hemerythrin was also co-expressed with hemoglobin in the sulfur-oxidizing symbiont-bearing trophosome tissue of the deep-sea hydrothermal vent tube worm *Ridgeia piscesae*, a polychaete annelid that is not closely related to *O. algarvensis* [58]. The function of hemerythrin in *Ridgeia* is at present unknown. It is intriguing that the two animals currently known to abundantly express both hemoglobin and hemerythrin, *O. algarvensis and R. piscesae*, live in symbiosis with sulfur-oxidizing bacteria.

### Interactions between the host innate immune system and its microbiome

We analyzed the proteins of the host innate immune system in our transcriptomes and proteomes, because these receptors, regulators and effectors are essential for sensing and responding to microbes [59], and are thus crucial for establishing and maintaining bacterial symbiosis [4]. The immune system must be able to distinguish beneficial symbionts from harmful intruders, and must respond appropriately, avoiding chronic inflammation in the presence of symbionts, while allowing rapid elimination of non-symbiotic bacteria.

#### Multitude of pattern recognition molecules for differential responses to microbes

Pattern recognition receptors (PRRs) are proteins that recognize microbe-associated molecular patterns (MAMPs) by binding to surface molecules specific to microbes, like peptidoglycan or lipopolysaccharide [60]. PRRs are essential for sensing the presence of different microbial species and initiating an appropriate response, either via activation of immune signaling pathways and the synthesis of antimicrobial compounds, or by dampening or silencing the immune response in the case of bacterial symbionts [4]. We identified many different types of classical pattern recognition receptors, as well as proteins potentially involved in pattern recognition via conserved domains (Table 1).

### PGRPs

Six peptidoglycan recognition proteins (OalgPGRP1-OalgPGRP6) were expressed in the *O. algarvensis* transcriptomes, and one of these was detected in the proteomes (OalgPGRP2, Table 1). PGRPs were first described as an important component of the innate immune defense [61], but are now known to play a major role in many animal-bacteria symbioses, mediating symbiont tolerance [62, 63], controlling symbiont populations [64], and regulating symbiosis establishment and maintenance [63, 65]. Elevated expression of PGRPs was also observed in the symbiont-bearing tissues of hydrothermal vent tube worms and mussels; however their precise function within these symbioses remains unknown [4, 66].

Specific PGRP function can not be determined from sequence information alone and depends on the molecular context in which they are expressed. However, some assumptions can be made and are discussed in the following. OalgPGRP1, OalgPGRP3 and OalgPGRP5 contained N-terminal transmembrane domains (indicating that they are membrane integral), as well as novel cytoplasmic domains (Fig. 2). As is typical for PGRPs, the poorly conserved cytoplasmic domains had no similarity to known sequences [34]. PGRPs that integrate into the cell membrane and carry intracellular domains often induce an antimicrobial response by activating immune signaling pathways like Toll and IMD (immune deficiency) [67, 68]. However, some PGRP receptors bind peptidoglycans, but do not pass on an intracellular signal, thus effectively down-regulating the immune response and mediating tolerance towards resident bacteria [69].

OalgPGRP2 and OalgPGRP4 consisted only of the conserved PGRP domain itself with a signal peptide, indicating that they are secreted (Fig. 2). Similar to the transmembrane PGRPs, secreted PGRPs can induce an antimicrobial response by indirectly activating immune signaling [70] or acting as bacterial growth inhibitors or antimicrobials themselves [71, 72]. However, if they possess amidase activity, they also can dampen the immune response, by cleaving peptidoglycan into non-immunogenic fragments [36, 73].

**Fig. 4:**
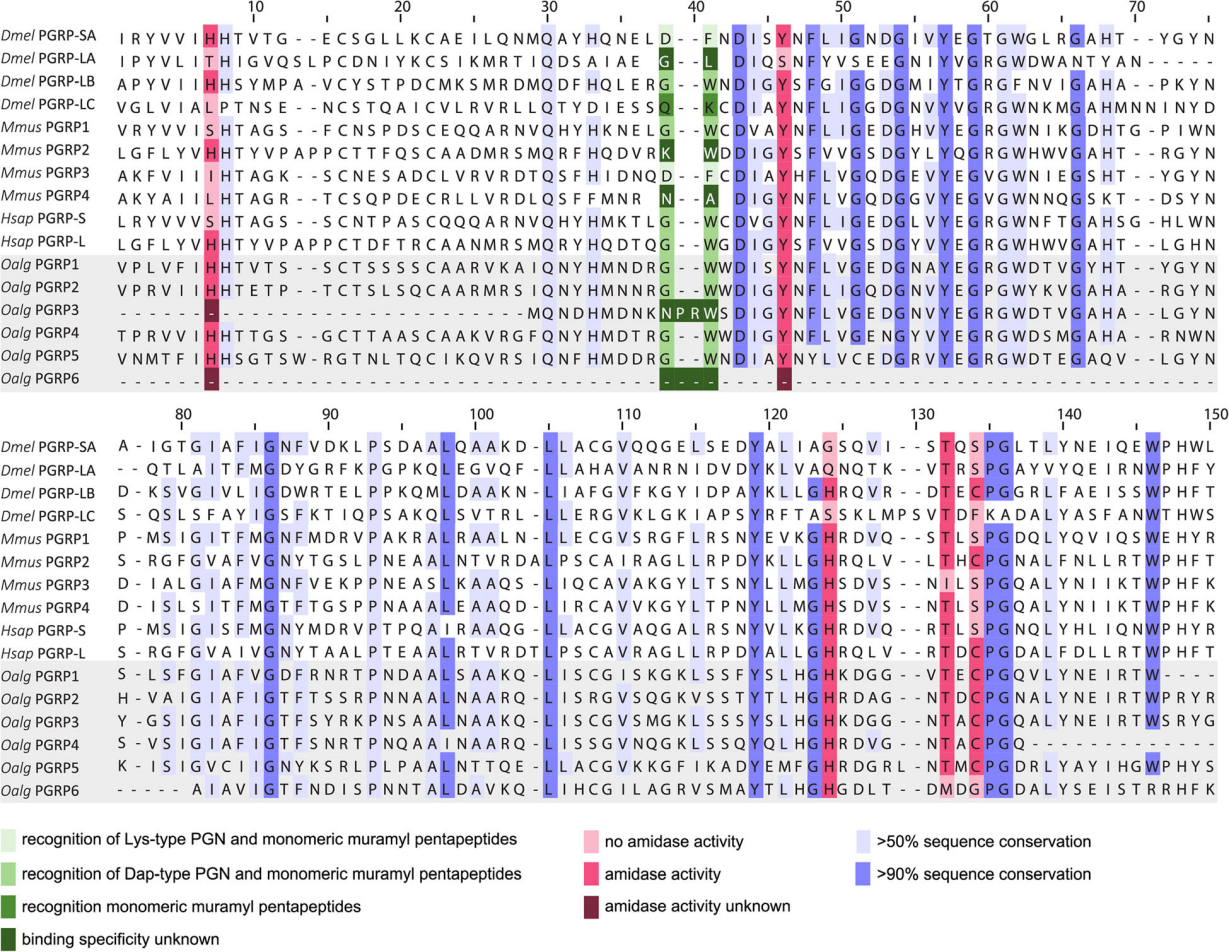
Protein alignment of peptidoglycan recognition proteins. Protein alignment of PGRP domain sequences from different model organisms and *Olavius algarvensis*; Dmel *Drosophila melanogaster* (GenBank accession numbers: PGRP-SA, Q9VYX7; PGRP-LA, Q95T64; PGRP-LB, Q8INK6; PGRP-LC, Q9GNK5), Mmus *Mus musculus* (GenBank accession numbers: PGRP1, O88593; PGRP2, Q8VCS0; PGRP3, A1A547; PGRP4, Q0VB07), Hsap *Homo sapiens* (GenBank accession numbers: PGRP-S, O75594; PGRP-L, Q96PD5), Oalg *Olavius algarvensis* (OalgPGRP1, comp330541_c4; OalgPGRP2, comp250229_c0; OalgPGRP3, comp335695_c10; OalgPGRP4, comp314994_c0; OalgPGRP5, comp332570_c2; OalgPGRP6, comp1100768_c0). Conserved active-site residues that confer amidase activity are shown in red; mutation of at least one active-site residue (pink) removes amidase activity

OalgPGRP1, OalgPGRP2, OalgPGRP4 and OalgPGRP5 contained the conserved residues needed to cleave peptidoglycan (Fig. 4 [36, 74]). This suggests that they contribute to symbiont tolerance by scavenging immunogenic peptidoglycan fragments, which are released as a by-product of bacterial growth. The sequence of OalgPGRP3 was incomplete, but contained four out of the five residues needed to cleave peptidoglycan (Fig. 4). These enzymatically active PGRPs may also play a role in symbiont population control and host nutrition by participating in the digestion of symbionts [75].

The affinities of PGRPs for different types of peptidoglycan stem peptides are determined by specific residues in the PGRP binding groove [76]. OalgPGRP1, OalgPGRP2, OalgPGRP4 and OalgPGRP5 possessed the residues that favor recognition of DAP-type peptidoglycan typical for gram negative bacteria [77], indicating that they could be used for the recognition of the worm's symbionts (which are all gram-negative) (Fig. 4). The specificity of OalgPGRP3 could not be assigned because it had an insertion of two amino acids in the binding-groove region, and the OalgPGRP6 fragment did not contain the binding-relevant region.

### Lectins

We detected six different classes of lectins in the transcriptome and proteome (Table 1, Table 4). They included C-type lectins, R-type lectins, fucolectin, SUEL/rhamnose-binding lectins, galectins, a beta-1,3-glucan binding protein and fibrinogen-like proteins. Lectins are proteins with widely differing molecular structures and physiological functions. They are unified by their ability to strongly, yet reversibly, bind specific carbohydrate residues on the surfaces of cells and proteins, without exhibiting enzymatic activity [78].

**Table 4:** Lectins expressed in *Olavius algarvensis*

Lectin group	Transcripts ^a)^	Proteins ^b)^	Domain architectures	Potential functions
*Fucolectins*	1	0		glycan recognition and host defense [126]
*C-type lectins*	33	5		glycan recognition [85], symbiont recognition and acquisition [127]
*R-type lectins*	6	4		glycan recognition [128], antimicrobial [129, 130], host defense [131]
*Galectins*	3	1		[132, 133], host defense [134, 135]
*SUEL rhamnose-binding lectins*	7	1		MAMP recognition [136, 137], egg fertilization [138], development [139]
*Fibrinogen-like proteins*	27	1		[92], MAMP recognition and antimicrobial activity [79], development [140], egg fertilization [141]

^a)^ Number of transcripts, defined as Trinity components; see [13]

^b)^ Number of identified proteins in the proteomes

Lectins are often associated with immune functions because of their molecular pattern recognition properties. For instance, they aid in microbe recognition and elimination through agglutination or direct antibacterial activity [79, 80], but, similar to PGRPs, are often also involved in modulating interactions between hosts and their beneficial symbionts. Lectins were, for example, shown to play major roles in symbiont acquisition and maintenance in sponges [81], corals [82, 83], clams [84], mice [85], and stilbonematid nematodes [86]. The sulfur-oxidizing symbionts of stilbonematine nematodes are very closely related to the primary symbionts of gutless oligochaetes [7, 87]. However, the stilbonematine lectins have no notable sequence similarity to the *O. algarvensis* lectins, as expected given the long independent evolutionary histories of these two animal groups [87].

The domain architectures of *Olavius* lectins and their potential functions in host-symbiont interaction are summarized in Table 4. C-type lectins were particularly diverse, and 33 different forms were found in the transcriptome. Some of these C-type lectins have significant sequence similarity to lectins implicated in host-microbe interactions (Additional file 1: Table S10), for example to CD209 antigen-like proteins, macrophage mannose receptors, and C-type lectin receptor B – all MAMP receptors and phagocytosis enhancers of bacteria in vertebrates [88–90], and to immunolectin A, a microbe-inducible C-type lectin in *Manduca sexta* (tobacco hornworm) that is also involved in phagocytosis [91] .

Another highly diverse group of lectins found in *O. algarvensis* were fibrinogen-related proteins (FREPs), which are almost exclusively involved in host-microbe interactions in invertebrates [92]. They were represented by 27 different unigenes (“components” in Trinity assembler terminology) in the transcriptome (Table 1, Table 4). For most of these, several isoforms with varying amino acid sequences were predicted, indicating that they may form an even more diverse array of proteins, possibly allowing very high specificity in the recognition of microbes.

### Scavenger receptor cysteine rich proteins

In the transcriptomes we found a large group of sequences containing single or tandem scavenger receptor cysteine rich (SRCR) domains, often in association with other conserved domains, such as C-type lectin, trypsin, epidermal growth factor, low density lipoprotein (LDL) receptor, and immunoglobulin domains (Additional file 1: Figure S3). One of these proteins, which contained an additional universal stress protein A and four LDL receptor class B domains, was also identified in the proteome (Table 1).

The SRCR domain is an ancient and highly conserved module often found in proteins of the innate immune system that are involved in the recognition of microbial patterns and phagocytosis of bacteria in vertebrates [93]. In invertebrates, SRCR proteins have been implicated in host-symbiont interaction [94] and MAMP recognition [95].

Many SRCR sequences we identified had significant similarity to the MARCO scavenger receptor, DMBT1, CD163/M130, sea urchin scavenger receptors, and lamprey Pema-SRCR protein (Additional file 1: Table S11); all of these proteins are known or have been implicated to be involved in immune functions [93, 96]. Similar to the *Olavius* FREPs, the SRCR sequences identified in the transcriptome were represented by a considerable number of unigenes (FREP: 27, SRCR: 25), but many more different isoforms were predicted by the assembly. We therefore expect a high variability in the final proteins, possibly supporting highly specific recognition of microbes in *Olavius*, as has been observed in other invertebrates [97].

### Toll-like receptors

We identified two Toll-like receptors (TLRs) consisting of the typical intracellular Toll/interleukin-1 receptor (TIR) homology domain and extracellular leucine- and cysteine-rich domains [98]. One of them was also detected in the proteome. Furthermore, we identified two sequences with only a TIR domain, one sequence with a TIR and transmembrane domain, and eight sequences containing leucine-rich repeats with high sequence similarity to TLRs from other animals and the variable lymphocyte receptors (VLRs) of agnate fish (Additional file 1: Table S12). VLRs are immune receptors that experience somatic recombination and convey a form of adaptive immunity in jawless vertebrates [99].

Toll-like receptors (TLRs) are microbial pattern recognition receptors and intracellular signaling transducers that play a vital role in sensing and responding to microbiota in many animals [100]. They also play a role in many beneficial host-microbe symbioses [101, 102]. TLRs have long been thought to be absent from annelids [103, 104]. However, their presence and importance in host-microbe interactions has recently been recognized in polychaetes, leeches and earthworms [105, 106], where some were shown to be involved in the innate immune response against pathogens [107, 108] or were constitutively expressed in the gut [109].

We identified all the major components of the Toll signaling pathway in *O. algarvensis*, indicating that Toll signaling is active (Additional file 1: Table S13). We identified SARM (sterile alpha and TIR motif containing protein), an inhibitor of Toll signaling [110], that could aid in down-regulating the immune response against symbionts. Tollip, another inhibitor of Toll signaling [111], was also detected in the proteome, suggesting that these two inhibitors of Toll signaling may protect *O. algarvensis* against constant inflammation in response to its symbionts.

### Interactions between symbionts and host may be regulated by different immune effectors and modulators

We detected several different types of antimicrobial proteins in the host transcriptome and proteome (Table 1), some of which were very abundant (Additional file 2: Table S8). The antimicrobials expressed in both transcriptome and proteome were lumbricin, an antimicrobial protein first discovered in earthworms [112], BPI (bactericidal permeability increasing protein), perforin/membrane attack complex-like proteins, insect defensin-like reeler proteins and cysteine-rich secretory proteins (Table 1). Antimicrobials combat infection by pathogenic microbes [113], but are also important in beneficial host-microbe interactions [85, 114], where they are used to modulate and control symbiont populations [115, 116]. In *O. algarvensis* they might be used to prevent symbionts and pathogens from invading non-symbiotic tissues, or to regulate symbiont growth.

## Conclusions

This study provides insights into the physiological and molecular mechanisms that allow *Olavius algarvensis* to live in a stable beneficial association with its microbial consortium. Our results indicate that these animals have undergone a number of evolutionary changes in adaptation to their symbiotic lifestyle, apart from a complete reduction of the excretory and digestive organs. Examples of such adaptations are host proteins involved in symbiont digestion and nutrient uptake, with likely relocalization of the expression sites of some of these enzymes, and unconventional proteins for gas exchange and storage.

Since a mouth and anus are absent in gutless oligochaetes, and their epidermis is covered by a thick layer of symbionts, foreign microbes can only invade these hosts if they have the ability to penetrate the egg integument, or the cuticle in a juvenile or adult worm, and pass through the symbiont layer just under the worm's cuticle. As a result, the complexity of the *O. algarvensis* microbiome is quite low and consists primarily of its five symbiotic phylotypes. Despite this low microbial diversity, we found that *O. algarvensis* expresses a highly diverse array of pattern recognition receptors, comparable to other invertebrates that are associated with a much more complex community of microbes on their skin and in their digestive system. The high number of MAMP recognition proteins expressed in the transcriptome and proteome that clearly originated from different genes demonstrate the need of *Olavius algarvensis* to differentially sense and respond to both its symbiotic microbiota as well as environmental bacteria, although direct contact with the latter may be limited. The transcriptomes generated in this study contained small amounts of contamination with other *Olavius* species (0.1 – 3.5%) and minor contaminations are also expected to be present in the proteomes. Therefore, transcripts and proteins with very low expression levels should be treated with caution, as they alternatively may have originated from closely related *Olavius* species. Particularly, if several variants of a transcript or protein were expressed, it is possible that some of the variants that were considerably less abundant than the most abundant variant could be derived from the contaminating species.

This is also the first comprehensive transcriptomic and proteomic analysis of the innate immune system of a marine oligochaete. It shows how genes common to a wide array of invertebrates have evolved to enable the intricate communication and interactions that occur between animals and their symbiotic microbiota. The analyses described here lay the foundation for future experimental studies of immune processes and physiological responses that are essential in the functioning of this symbiosis.

## Additional files

Additional file 1: Table S1. Summary of transcriptome sequencing. **Table S2**. Summary of transcriptome assembly and protein database. **Table S3**. Total protein of symbionts from fresh compared to starved whole worms. **Table S4**. Expression patterns of typical intestinal proteases in other animals. **Table S5**. High similarity of host digestive proteins to earthworm midgut enzymes. **Table S6**. Similarity of host digestive glucosidases to intestinal glucosidases of other animals. **Table S7**. *O. algarvensis* giant extracellular hemoglobin sequences. **Table S9**. Hemerythrin expression in annelids. **Table S10**. Host c-type lectins with similarity to known immune lectins. **Table S11**. Host SRCR proteins with similarity to SRCR proteins involved in immune processes. **Table S12**. Host Toll-like receptor sequences. **Table S13**. Toll immune signaling pathway in *Olavius.* **Figure S1**. Transcriptome annotation statistics. **Figure S2**: Hydrophobicity cluster analysis plots of annelid hemoglobin chains. **Figure S3**. Domain structures of proteins with scavenger domains. (DOCX 920 kb)
Additional file 2: Table S8. Proteins potentially involved in symbiont interaction in *O. algarvensis*, all transcripts and proteins. (XLSX 92 kb)
Additional file 3: Table S14. Subcellular localization evidence of host digestive proteins. (XLSX 17 kb)

## Abbreviations

AMP: Antimicrobial protein/peptide
cDNA: Complementary DNA
CDS: Coding DNA sequence
CO: Carbon monoxide
FREP: Fibrinogen related protein
PGRP: Peptidoglycan recognition protein
SRCR: Scavenger receptor cysteine-rich
TLR: Toll-like receptor
