# Supplementary material for: Transcriptomic and proteomic insights into innate immunity and adaptations to a symbiotic lifestyle in the gutless marine worm Olavius algarvensis
Source: BMC Genomics. 2016 Nov 21;17:942. doi: 10.1186/s12864-016-3293-y (PMC5117596; doi:10.1186/s12864-016-3293-y)
Supplement: Additional file 1: Table S1. — Summary of transcriptome sequencing. Table S2. Summary of transcriptome assembly and protein database. Table S3. Total protein of symbionts from fresh compared to starved whole worms. Table S4. Expression patterns of typical intestinal proteases in other animals. Table S5. High similarity of host digestive proteins to earthworm midgut enzymes. Table S6. Similarity of host digestive glucosidases to intestinal glucosidases of other animals. Table S7. O. algarvensis giant extracellular hemoglobin sequences. Table S9. Hemerythrin expression in annelids. Table S10. Host c-type lectins with similarity to known immune lectins. Table S11. Host SRCR proteins with similarity to SRCR proteins involved in immune processes. Table S12. Host Toll-like receptor sequences. Table S13. Toll immune signaling pathway in Olavius. Figure S1. Transcriptome annotation statistics. Figure S2: Hydrophobicity cluster analysis plots of annelid hemoglobin chains. Figure S3. Domain structures of proteins with scavenger domains. (DOCX 920 kb) [file 12864_2016_3293_MOESM1_ESM.docx]

## Additional file Table 1: Summary of transcriptome sequencing

Library A was prepared from whole worms freshly collected from the environment. Library B was prepared from whole worms after anoxic incubation (see *Experimental Procedures* for details).

| **Sequencing statistic** | **library A** | **library B** |
| --- | --- | --- |
| number of read pairs | 167,402,116 | 6,041,419 |
| read length (bp) | 100 | 100 |
| total bases | 33,815,227,432 | 1,196,200,962 |
| number of read pairs after quality processing | 159,551,509 | 5,745,537 |
| total bases filtered/trimmed reads | 26,801,302,242 | 987,624,581 |

## Additional file Table 2: Summary of transcriptome assembly and protein database

| **Transcriptome assembly** |  |
| --- | --- |
| number of contigs | 173,602 |
| number of contigs >= 500 bp | 60,369 |
| number of contigs >= 1000 bp | 23,719 |
| N50 | 1236 |
| total size of assembly (bp) | 100,372,073 |
| number of contigs with blast hits (blastx vs ncbi nr, e-value cut-off 1e-6) | 40,860 |
| number of contigs with interpro hits (interproscan*) | 137,596 |
| **CDS prediction** |  |
| predicted non-redundant CDS (getorf) after 99% identity clustering | 1,306,981 |
| predicted CDS (FrameDP) after 99% identity clustering | 54,909 |
| Final non-redundant host sequences | 1,359,455 |

* Databases searched: ProDom [<corpet1998>], Prints [<attwood1994>], PIR [<wu2002>], Pfam [<finn2008>], Smart [<letunic2004>], TIGRFAM [<haft2003>], PROSITE [<hulo2006>], HAMAP [8], SuperFamily [<wilson2007>], SignalP [<petersen2011>], TMHMM [<krogh2001>], Panther [<mi2005>], Gene3D [<pearl2005>], Phobius [<kaell2004>], Coils [<lupas1991>]

## Additional file Table 3: Total protein of symbionts from fresh compared to starved whole worms

Listed are nSpC (normalized spectral counts) of proteins assigned to each *O. algarvensis* symbiont. Proteins were assigned as described in [<kleiner2012>] to each symbiont species based on the symbiont genomes as published in [<woyke2006>]. Each sample corresponds to one biological replicate sample of *O. algarvensis* worms as described in the *Experimental Procedures* section of this paper. Fresh worm samples were prepared from freshly collected, untreated material, while starved worm samples were prepared from worms that had been incubated for 8 days under oxic conditions without external energy sources that would allow the symbionts to fix carbon and grow. Significant differences between fresh and starved samples were determined with a Student's t-Test (significant values marked “*”). Rep, technical replicate; n.d., not determined.

| protein abundance whole worms fresh | sample 1 nSpC | | sample 2 nSpC | | sample 3 nSpC | |  |
| --- | --- | --- | --- | --- | --- | --- | --- |
|  | rep. 1 | rep. 2 | rep. 1 | rep. 2 | rep. 1 | rep. 2 | average nSpC |
| total symbiont | 2682.47 | 2609.84 | 3016.81 | n.d. | 3487.93 | n.d. | 2949.26 |
| total *OalgG1* | 775.99 | 784.99 | 714.70 | n.d. | 780.23 | n.d. | 763.98 |
| total *OalgG3* | 107.02 | 109.42 | 110.08 | n.d. | 120.44 | n.d. | 111.74 |
| total *OalgD1* | 36.20 | 33.61 | 27.08 | n.d. | 39.52 | n.d. | 34.10 |
| total *OalgD4* | 12.19 | 18.24 | 42.50 | n.d. | 57.43 | n.d. | 32.59 |
| total unclassified symbionts | 1750.65 | 1662.39 | 2122.45 | n.d. | 2490.31 | n.d. | 2006.45 |
|  |  |  |  |  |  |  |  |
| protein abundance whole worms starved | sample 1 nSpC | | sample 2 nSpC | | sample 3 nSpC | |  |
|  | rep 1 | rep 2 | rep 1 | rep 2 | rep 1 | rep 2 | average nSpC |
| total symbiont | 1737.92 | 1724.54 | 1859.96 | 1968.93 | 1790.71 | 2136.52 | 1869.76 |
| total *OalgG1* | 385.41 | 391.17 | 446.44 | 474.63 | 437.98 | 492.11 | 437.96 |
| total *OalgG3* | 60.82 | 73.21 | 84.22 | 101.28 | 80.19 | 78.99 | 79.79 |
| total *OalgD1* | 16.28 | 39.66 | 36.06 | 23.84 | 43.64 | 65.27 | 37.46 |
| total *OalgD4* | 28.06 | 47.39 | 45.46 | 53.41 | 34.83 | 35.26 | 40.74 |
| total unclassified symbionts | 1247.35 | 1172.44 | 1247.78 | 1315.77 | 1193.79 | 1464.89 | 1273.67 |
|  |  |  |  |  |  |  |  |
| protein abundance whole worms | average nSpC fresh worms | | | average nSpC starved worms | | |  |
|  | sample 1 | sample 2 | sample 3 | sample 1 | sample 2 | sample 3 | p-value t-test |
| total symbiont | 2646.16 | 3016.81 | 3487.93 | 1731.23 | 1914.45 | 1963.62 | 0.00963* |
| total *OalgG1* | 780.49 | 714.70 | 780.23 | 388.29 | 460.54 | 465.05 | 0.00064* |
| total *OalgG3* | 108.22 | 110.08 | 120.44 | 67.02 | 92.75 | 79.59 | 0.01655* |
| total *OalgD1* | 34.91 | 27.08 | 39.52 | 27.97 | 29.95 | 54.46 | 0.71551* |
| total *OalgD4* | 15.22 | 42.50 | 57.43 | 37.73 | 49.44 | 35.05 | 0.86642* |
| total unclassified symbionts | 1706.52 | 2122.45 | 2490.31 | 1209.90 | 1281.78 | 1329.34 | 0.02205* |

## Additional file Table 4: Expression patterns of typical intestinal proteases in other animals

List of digestive enzymes found in *Olavius algarvensis* and their typical expression location in other animals

| **enzyme name** | **species** | **common name** | **tissue expression** | **reference** |
| --- | --- | --- | --- | --- |
| pancreatic carboxypeptidase A1 | *Bos taurus* | cattle | pancreas | [<petra1971>] |
|  | *Homo sapiens* | human | pancreas | [<catasus1992>] |
|  | *Rattus norvegicus* | rat | pancreas | [<quinto1982>] |
| pancreatic carboxypeptidase A2 | *Homo sapiens* | human | pancreas | [<catasus1995>] |
|  | *Rattus norvegicus* | rat | pancreas | [<gardell1988>] |
| carboxypeptidase A | *Aedes aegypti* | mosquito | gut | [<edwards2000>] |
|  | *Helicoverpa armigera* | corn earworm | midgut | [<bown1998>] |
|  | *Lutzomyia longipalpis* | sand fly | midgut | [<jochim2008>] |
|  | *Rhodnius prolixus* | kissing bug | gut | [<ribeiro2014>] |
|  | *Tenebrio molitor* | yellow mealworm | gut | [<prabhakar2007>] |
|  | *Trichoplusia ni* | cabbage looper | midgut | [<wang2004>] |
| zinc carboxypeptidase | *Eisenia andrei* | earthworm | midgut | [<lee2005>] |
| chymotrypsin A | *Bos taurus* | cattle | pancreas | [<hartley1964>] |
|  | *Gadus morhua* | atlantic cod | gut | [<asgeirsson1991>] |
| chymotrypsin B | *Canis lupus* | dog | pancreas | [<pinsky1983>] |
|  | *Gadus morhua* | atlantic cod | pyloric caeca | [<spilliaert2000>] |
|  | *Homo sapiens* | human | pancreas | [<tomita1989>] |
|  | *Rattus norvegicus* | rat | pancreas | [<bell1984>] |
| chymotrypsin | *Agrotis ipsilon* | black cutworm | midgut | [<mazumdar-leighton2001>] |
|  | *Haliotis rufescens* | red abalone | digestive gland | [<degnan1995>] |
|  | *Helicoverpa armigera* | corn earworm | gut | [<chougule2005>] |
|  | *Helicoverpa zea* | corn earworm | midgut | [<mazumdar-leighton2001>] |
|  | *Heliothis virescens* | tobacco budworm | midgut | [<zhu2012>] |
|  | *Mayetiola destructor* | hessian fly | midgut | [<zhu2005>] |
|  | *Sepia officinalis* | cuttlefish | hepatopancreas | [<balti2012>] |
|  | *Tenebrio molitor* | yellow mealworm | midgut | [<elpidina2005>] |
| chymotrypsin-like protease ctrl-1 | *Homo sapiens* | human | pancreas | [<reseland1997>] |
| pancreatic elastase | *Homo sapiens* | human | pancreas | [<largman1976>] |
|  | *Manduca sexta* | tobacco hawkmoth | midgut | uniprot accession: Q25510 |
|  | *Rattus norvegicus* | rat | pancreas | [<largman1983>] |
|  | *Sus scrofa* | pig | pancreas | [<gertler1970>] |
| cathepsin B | *Eisenia andrei* | earthworm | midgut | [<lee2005>] |
|  | *Haliotis discus hannai* | Pacific abalone | digestive gland | [<qiu2013>] |
|  | *Litopenaeus vannamei* | white shrimp | midgut | [<stephens2012>] |
|  | *Meretrix meretrix* | Asiatic hard clam | digestive gland | [<wang2008>] |
|  | *Necator americanus* | human hookworm | gut | [<ranjit2008>] |
|  | *Opisthorchis viverrini* | human liver fluke | gut | [<pinlaor2009>] |
|  | *Pandalus borealis* | northern shrimp | hepatopancreas | [<aoki2003>] |
|  | *Tityus serrulatus* | scorpion | midgut | [<fuzita2015>] |
| cathepsin F | *Clonorchis sinensis* | Chinese liver fluke | intestine | [<kang2010>] |
|  | *Homo sapiens* | human | ubiquitous | [<wang1998>] |
|  | *Mus musculus* | mouse | ubiquitous | [<deussing2000>] |
|  | *Paralichthys olivaceus* | olive flounder | ubiquitous | [<ahn2009>] |
|  | *Tityus serrulatus* | scorpion | midgut | [<fuzita2015>] |
| cathepsin L | *Cristaria plicata* | cockscomb pearl mussel | hepatopancreas | [<hu2014>] |
|  | *Drosophila melanogaster* | fruit fly | midgut | [<matsumoto1995>] |
|  | *Eriocheir sinensis* | Chinese mitten crab | hepatopancreas | [<li2010>] |
|  | *Homo sapiens* | human | ubiquitous | [<gal1988>] |
|  | *Metapenaeus ensis* | greasyback shrimp | hepatopancreas | [<hu2007>] |
|  | *Mus musculus* | mouse | ubiquitous | [<portnoy1986>] |
|  | *Pinctada fucata* | pearl oyster | digestive gland | [<ma2010>] |
|  | *Rattus norvegicus* | rat | ubiquitous | [<ishidoh1987>] |
|  | *Tenebrio molitor* | yellow mealworm | midgut | [<cristofoletti2005>] |
|  | *Tityus serrulatus* | scorpion | midgut | [<fuzita2015>] |

## Additional file Table 5: High similarity of host digestive proteins to earthworm midgut enzymes

*Olavius algarvensis* digestive enzymes were blasted (tblastn) against the only available gut-specific EST library of an oligochaete, the earthworm *Eisenia andrei*; n.d., not detected; n.a., not available, aa, amino acid.

| **transcript ID ^a)^** | **protein ID** | **annotation** | **FPKM ^b)^** | **Proteome ^c)^** | **Accession ^d)^** | **e-value** | **aa similarity** |
| --- | --- | --- | --- | --- | --- | --- | --- |
| ***Protein digestion*** |  |  |  |  |  |  |  |
| comp330196_c1 | n.d. | carboxypeptidase a2 | 8.95 | n.d. | BP524493.1 | 1.16E-60 | 68% |
| comp310626_c3 | n.d. | carboxypeptidase a1 | 4.70 | n.d. | BP524493.1 | 3.21E-20 | 62% |
| comp209868_c0 | n.d. | carboxypeptidase a2 | 0.53 | n.d. | no hit | n.d. | n.d. |
| comp329532_c3 | n.d. | carboxypeptidase a2 | 7.24 | n.d. | BP524493.1 | 2.34E-49 | 66% |
| comp328734_c12 | n.d. | carboxypeptidase | 1.98 | n.d. | no hit | n.d. | n.d. |
| comp328734_c1 | n.d. | carboxypeptidase | 0.00 | n.d. | BP524487.1 | 7.13E-10 | 59% |
| comp326419_c0 | n.d. | carboxypeptidase | 14.89 | n.d. | BP524487.1 | 1.64E-28 | 54% |
| comp330196_c2 | n.d. | carboxypeptidase | 8.49 | n.d. | BP524771.1 | 7.1E-9 | 65% |
| comp319717_c3 | n.d. | lysosomal Pro-X carboxypeptidase | 0.31 | n.d. | no hit | n.d. | n.d. |
| comp320275_c1 | n.d. | chymotrypsin A | 10.47 | n.d. | no hit | n.d. | n.d. |
| comp306409_c1 | n.d. | chymotrypsin B | 1.60 | n.d. | BP524415.1 | 9.96E-11 | 67% |
| comp334148_c2 | BF11_334148_c2_seq1_11 BF11_334148_c2_seq2_10 | chymotrypsin-like protease ctrl-1 | 29.94 | 4.69 | BP524722.1 | 1.28E-14 | 65% |
| comp331491_c1 | BF11_331491_c1_seq1_7 | pancreatic elastase | 14.03 | 3.92 | BP524414.1 | 1.57E-10 | 55% |
| comp334775_c2 | n.d. | cathepsin B | 1.96 | n.d. | no hit | n.d. | n.d. |
| comp335560_c0 | BF11_335560_c0_seq5_5 BF11_335560_c0_seq9_5 | cathepsin B | 355.52 | 1.03 | BP524429.1 | 9.23E-11 | 70% |
| comp306522_c2 | n.d. | cathepsin F | 0.82 | n.d. | no hit | n.d. | n.d. |
| comp306522_c3 | n.d. | cathepsin F | 5.83 | n.d. | no hit | n.d. | n.d. |
| comp308536_c0 | BF11_308536_c0_seq1_12 | cathepsin F | 126.33 | 0.03 | no hit | n.d. | n.d. |
| comp283346_c1 | n.d. | cathepsin L | 0.19 | n.d. | no hit | n.d. | n.d. |
| comp306922_c1 | BF11_306922_c1_seq1_34 | cathepsin L | 498.41 | 1.87 | BP524408.1 | 3.86E-53 | 83% |
| comp315575_c0 | n.d. | cathepsin L | 20.01 | n.d. | no hit | n.d. | n.d. |
| comp315575_c2 | FD_315575_c2_seq1:64:270:1:+ | cathepsin L | 39.31 | n.d. | no hit | n.d. | n.d. |
| comp328653_c0 | n.d. | cathepsin L | 10.39 | n.d. | BP524408.1 | 6.31E-43 | 78% |
| comp329800_c6 | n.d. | cathepsin O | 4.58 | n.d. | BP524408.1 | 9.79E-7 | 51% |
| comp314408_c1 | n.d. | cathepsin Z | 0.79 | n.d. | no hit | n.d. | n.d. |
| ***Carbohydrate digestion*** | |  |  |  |  |  |  |
| comp328746_c4 | n.d. | alpha amylase | 2.71 | n.d. | no hit | n.d. | n.d. |
| comp324906_c1 | BF11_324906_c1_seq1_9 BF11_324906_c1_seq2_12 | alpha amylase | 25.14 | 0.00 | no hit | n.d. | n.d. |
| comp335205_c1 | BF11_335205_c1_seq1_20 | lysosomal alpha glucosidase | 30.77 | 2.91 | no hit | n.d. | n.d. |
| comp320084_c0 | n.d. | lysosomal beta-mannosidase | 4.26 | n.d. | no hit | n.d. | n.d. |
| comp334411_c3 | n.d. | sucrase-isomaltase | 0.00 | n.d. | no hit | n.d. | n.d. |
| comp329957_c8 | BF11_329957_c8_seq2_17 | acid trehalase | 20.77 | 0.03 | no hit | n.d. | n.d. |
| comp335402_c7 | n.d. | acid trehalase | 3.32 | n.d. | no hit | n.d. | n.d. |
| ***Lipid degradation*** |  |  |  |  |  |  |  |
| comp22535_c0 | n.d. | lysosomal acid lipase | 1.34 | n.d. | no hit | n.d. | n.d. |
| ***Peptidoglycan degradation*** | |  |  |  |  |  |  |
| comp249291_c0 | n.d. | lysozyme | 460.97 | n.d. | BP524379.1 | 3.00E-28 | 78% |
| comp250229_c0 | BF11_250229_c0_seq1_15 BF11_250229_c0_seq2_17 | peptidoglycan recognition protein | 180.88 | n.d. | BP524394.1 | 8.43E-35 | 66% |
| comp335695_c10 | n.d. | peptidoglycan recognition protein | 123.41 | n.d. | BP524394.1 | 3.12E-25 | 69% |
| comp330541_c4 | n.d. | peptidoglycan recognition protein | 43.36 | n.d. | BP524394.1 | 4.86E-35 | 70% |
| comp314994_c0 | n.d. | peptidoglycan recognition protein | 7.11 | n.d. | BP524394.1 | 3.09E-32 | 66% |
| comp332570_c2 | n.d. | peptidoglycan recognition protein | 21.15 | n.d. | BP524394.1 | 1.35E-31 | 68% |

^a)^ Defined as trinity components, see [<grabherr2011>]

^b)^ Average transcript abundance in transcriptomes

^c)^ Average protein abundance in proteomes in ^0^/_000_

^d)^ GenBank accession numbers (NCBI EST)

## Additional file Table 6: Similarity of host digestive glucosidases to intestinal glucosidases of other animals

| **transcript ID** | **conserved domains** | **annotation** | **database** | **Blast Hit Description (HSP)** | **organism** | **NCBI accession** | **e-value** | **aa sim.** |
| --- | --- | --- | --- | --- | --- | --- | --- | --- |
| comp329957_c8_seq1 | glycoside hydrolase family 65 (PF03632) trehalose and maltose hydrolase (COG1554) | acid trehalase | ncbi nr | maltose phosphorylase | *Culex quinquefasciatus* | XP_001850772.1 | 1,41E-68 | 48% |
|  |  |  | ncbi nr | acid trehalase-like protein 1 | *Homo sapiens* | NP_079368.3 | 1,41E-68 | 49% |
|  |  |  | swissprot | acid trehalase-like protein 1 | *Homo sapiens* | Q32M88 | 1,00E-77 | 29% |
| comp329957_c8_seq2 | glycoside hydrolase family 65 (PF03632) trehalose and maltose hydrolase (COG1554) | acid trehalase | ncbi nr | maltose phosphorylase | *Culex quinquefasciatus* | XP_001850772.1 | 1,56E-68 | 48% |
|  |  |  | ncbi nr | acid trehalase-like protein 1 | *Homo sapiens* | NP_079368.3 | 1,56E-68 | 49% |
|  |  |  | swissprot | acid trehalase-like protein 1 | *Homo sapiens* | Q32M88 | 1,00E-77 | 29% |
| comp335402_c7_seq1 | glycoside hydrolase family 65 (PF03632) | acid trehalase | ncbi nr | acid trehalase-like protein 1 | *Danio rerio* | NP_001071193.1 | 1,93E-61 | 66% |
|  |  |  | ncbi nr | acid trehalase-like protein 1 | *Mus musculus* | NP_663362.2 | 1,30E-57 | 63% |
|  |  |  | swissprot | acid trehalase-like protein 1 | *Danio rerio* | A0JMP0 | 3,00E-75 | 48% |
| comp334411_c3_seq1 | alpha-glucosidase (PTHR22762) | sucrase-isomaltase | ncbi nr | sucrase-isomaltase | *Mus musculus* | ACH86012.1 | 4,12E-07 | 61% |
|  |  |  | swissprot | glucoamylase | *Homo sapiens* | Q2M2H8 | 5,00E-08 | 41% |
|  |  |  | swissprot | sucrase-isomaltase, intestinal | *Suncus murinus* | O62653 | 2,00E-07 | 40% |
| comp328746_c4_seq1 | alpha amylase catalytic domain family (cl07893) | pancreatic alpha-amylase | ncbi nr | amylase, alpha 2A, pancreatic | *Danio rerio* | AAH62867.1 | 1,26E-19 | 70% |
|  |  |  | swissprot | salivary alpha-amylase | *Homo sapiens* | P04745 | 7,00E-22 | 56% |
| comp324906_c1_seq1 | alpha amylase, catalytic domain (PFAM00128) | salivary alpha-amylase | ncbi nr | alpha-amylase | *Aedes aegypti* | XP_001656785.1 | 4,27E-80 | 50% |
|  |  |  | ncbi nr | salivary alpha-glucosidase | *Culex tarsalis* | ACJ64288.1 | 9,22E-75 | 53% |
|  |  |  | swissprot | probable maltase | *Aedes aegypti* | P13080 | 6,00E-85 | 34% |
| comp324906_c1_seq2 | alpha amylase, catalytic domain (PFAM00128) | salivary alpha-amylase | ncbi nr | alpha-amylase | *Aedes aegypti* | XP_001656785.1 | 1,35E-89 | 51% |
|  |  |  | ncbi nr | salivary alpha-glucosidase | *Culex tarsalis* | ACJ64288.1 | 4,67E-82 | 54% |
|  |  |  | swissprot | probable maltase | *Aedes aegypti* | P13080 | 8,00E-89 | 35% |

## Additional file Table 7: *O. algarvensis* giant extracellular hemoglobin sequences

Table lists details on giant hemoglobin (globin and linker) chains identified in *Olavius algarvensis,* n.d. not detected.

| **transcript component ID ^a)^** | **protein ID** | **annotation** | **signalP ^b)^** | **conserved domains** | **FPKM ^c)^** | **Proteome ^d)^** |
| --- | --- | --- | --- | --- | --- | --- |
| comp254846_c0 | BF11_254846_c0_seq1_18 | hemoglobin chain | no | globin IPR000971 | 39.05 | 1.31 |
| comp287449_c0 | BF11_287449_c0_seq1_4 | hemoglobin chain | yes | none | 911.55 | 32.18 |
| comp300058_c0 | n.d. | hemoglobin chain | yes | globin IPR000971 | 6.40 | n.d. |
| comp300058_c1 | n.d. | hemoglobin chain | no | globin IPR000971 | 6.65 | n.d. |
| comp307848_c0 | BF11_307848_c0_seq1_17 | hemoglobin chain | yes | none | 980.87 | 31.07 |
| comp309954_c0 | n.d. | hemoglobin chain | no | globin IPR000971 | 22.33 | n.d. |
| comp321227_c0 | BF11_321227_c0_seq1_9 | linker chain | yes | LDL receptor class A repeat IPR002172 | 726.5 | 2.46 |
| comp321285_c0 | BF11_321285_c0_seq1_16, BF11_321285_c0_seq2_7 | hemoglobin and linker chains | yes | globin IPR000971 | 5679.8 | 41.16 |
| comp324803_c0 | BF11_324803_c0_seq1_26 | linker chain | no | none | 400.82 | 4.35 |
| comp326155_c0 | n.d. | linker chain | no | LDL receptor class A repeat IPR002172 | 3.51 | n.d. |
| comp327543_c0 | BF11_327543_c0_seq1_6 | hemoglobin chain | no | none | 2.84 | 5.59 |
| comp327543_c2 | BF11_327543_c2_seq1_2 | hemoglobin chain | yes | globin IPR000971 | 3.75 | 0.26 |
| comp327754_c0 | BF11_327754_c0_seq1_26 | hemoglobin chain | yes | globin IPR000971 | 2289.10 | 36.27 |
| comp328129_c0 | BF11_328129_c0_seq1_1 | hemoglobin chain | yes | globin IPR000971 | 38.25 | 1.52 |
| comp331953_c0 | BF11_331953_c0_seq1_4 | linker chain | no | LDL receptor class A repeat IPR002172 | 64.48 | 0.00 |
| comp332275_c3 | BF11_332275_c3_seq1_14 | hemoglobin chain | no | globin IPR000971 | 4.37 | 2.23 |
| comp335909_c2 | BF11_335909_c2_seq2_7 | linker chain | no | LDL receptor class A repeat IPR002172 | 5.86 | 0.00 |

^a)^ Defined as trinity components; see [<grabherr2011>]

^b)^ Presence/absence of signal peptide as determined with SignalP [<petersen2011>]

^c)^ Average transcript abundance in transcriptomes

^d)^ Average protein abundance in proteomes in ^0^/_000_

## Additional file Table 8: Proteins potentially involved in symbiont interaction in *O. algarvensis*, all transcripts and proteins

Table is too large; refer to supplementary file “Supplementary_Table8.xlsx”

## Additional file Table 9: Hemerythrin expression in annelids

Out of 67 annelid species for which EST data is available in the public databases, hemerythrin was only expressed in the seven species listed here.

| **species** | **class/subclass** | **library name** | **#hemerythrin ESTs/total ESTs** | **accession** |
| --- | --- | --- | --- | --- |
| *Lumbricus rubellus* | Oligochaeta | LIBEST_007207 Earthworm Lambda Zap Express Library (whole worm adult) | 2/1925 | EL517735.1, EL517681.1 |
|  |  | LIBEST_014450 Earthworm Cadmium Exposure Library (whole worm adult) | 2/2230 | DR077506.1, DR077413.1 |
|  |  | LIBEST_015953 Earthworm Head Enriched library (adult) | 5/2569 | DR077018.1, DR076532.1, DR076454.1, DR076360.1, DR075863.1 |
|  |  | LIBEST_017401 Earthworm Copper Exposure Library (whole worm adult) | 2/1518 | DR009706.1, DR009119.1 |
|  |  | LIBEST_016202 Juvenile Earthworm Library (whole worm juvenile) | 1/2895 | CV072407.1 |
|  |  | LIBEST_014289 Lumbricus rubellus Late Cocoon Library 1 (whole worm late cocoon) | 14/2728 | CO046674.1, CO046632.1, CF839199.1, CF810104.1, CF809646.1, CF809632.1, CF426790.2, CF416883.2, CF416571.2, CF416434.2, CF416392.2, CF415962.2, CF415927.2, CF416875.1 |
| *Perionyx excavatus* | Oligochaeta | LIBEST_022800 Perionyx excavatus regenerating tissue cDNA library (head regeneration, adult worms) | 8/1195 | BP998686.1, BP998685.1, BP998684.1, BP998683.1, BP998682.1, BP998681.1, BP998680.1, BP998679.1 |
| *Amynthas koreanus* | Oligochaeta | LIBEST_026527 Amynthas koreanus whole body cDNA library | 3/2150 | FS507482.1, FS507353.1, FS507056.1 |
| *Eisenia fetida* | Oligochaeta | LIBEST_026326 Earthworm SSH cDNA library (*E. coli* challenged adults, whole worms) | 2/394 | HO001559.1, HO001494.1 |
| *Haementeria depressa* | Hirudinea | LIBEST_015769 Leech Haementeria depressa library HDA (salivary gland of adult worms) | 18/891 | CN807659.1, CN807658.1, CN807657.1, CN807656.1, CN807655.1, CN807654.1, CN807653.1, CN807652.1, CN807651.1, CN807650.1, CN807649.1, CN807648.1, CN807647.1, CN807646.1, CN807645.1, CN807644.1, CN807643.1, CN807642.1 |
| Macrobdella decora | Hirudinea | LIBEST_028114 North American medicinal leech Macrobdella decora salivary gland library | 4/1604 | JZ187845.1, JZ186990.1, JZ186989.1, JZ186988.1 |
| *Riftia pachyptila* | Polychaeta | symbiont-bearing trophosome (TR-BW) | 5/59 | [<sanchez2007>] |

## Additional file Table 10: Host c-type lectins with similarity to known immune lectins

| **transcript ID** | **NCBI Accession** | **Blast Hit Description (HSP)** | **organism** | **e-value** | **aa similarity** |
| --- | --- | --- | --- | --- | --- |
| comp289680_c0_seq1 | NP_001178934.1 | CD209 antigen-like protein E | *Rattus norvegicus* | 7.45E-011 | 48% |
|  | AAI07190.1 | CD209e antigen | *Mus musculus* | 9.73E-011 | 46% |
|  | NP_507557.1 | C-type Lectin family member (clec-42) | *Caenorhabditis elegans* | 1.27E-010 | 46% |
| comp292689_c0_seq1 | XP_221790.4 | CD209 molecule-like | *Rattus norvegicus* | 4.95E-008 | 62% |
| comp329897_c1_seq1 | NP_001186302.1 | CD209 molecule | *Danio rerio* | 2.96E-008 | 53% |
|  | NP_002429.1 | macrophage mannose receptor 1 | *Homo sapiens* | 6.60E-008 | 45% |
| comp335421_c2_seq1 | CAZ65474.1 | C-type Lectin | *Caenorhabditis elegans* | 1.58E-008 | 36% |
|  | NP_001179599.1 | C-type mannose receptor 2 | *Bos taurus* | 6.02E-008 | 42% |
| comp335421_c2_seq2 | NP_001179599.1 | C-type mannose receptor 2 | *Bos taurus* | 6.16E-008 | 42% |
|  | NP_006030.2 | C-type mannose receptor 2 | *Homo sapiens* | 1.37E-007 | 41% |
| comp335421_c2_seq6 | NP_001179599.1 | C-type mannose receptor 2 | *Bos taurus* | 4.52E-008 | 42% |
|  | NP_006030.2 | C-type mannose receptor 2 | *Homo sapiens* | 1.01E-007 | 41% |
| comp335598_c3_seq2 | ADD13530.1 | IML1 immunolectin 1 | *Manduca sexta* | 9.14E-010 | 50% |
|  | AAC33576.1 | immunolectin-A precursor | *Manduca sexta* | 9.14E-010 | 50% |
| comp336008_c0_seq13 | ABW34402.1 | immunity adhesion receptor L-SIGN | *Nomascus leucogenys* | 7.97E-014 | 52% |
|  | AAR04559.1 | L-SIGN variant | *Homo sapiens* | 1.15E-012 | 50% |
| comp336008_c0_seq15 | NP_001178934.1 | CD209 antigen-like protein E | *Rattus norvegicus* | 2.50E-012 | 52% |
|  | ABW34402.1 | immunity adhesion receptor L-SIGN | *Nomascus leucogenys* | 2.11E-011 | 51% |
|  | AAR04559.1 | L-SIGN variant | *Homo sapiens* | 4.71E-011 | 50% |
| comp336008_c0_seq6 | NP_001178934.1 | CD209 antigen-like protein E | *Rattus norvegicus* | 1.18E-012 | 50% |
|  | ABW34402.1 | immunity adhesion receptor L-SIGN | *Nomascus leucogenys* | 1.70E-011 | 51% |
|  | AAR04559.1 | L-SIGN variant | *Homo sapiens* | 3.79E-011 | 50% |

## Additional file Table 11: Host SRCR proteins with similarity to SRCR proteins involved in immune processes

| **NCBI accession** | **transcript** | **Blast Hit Description (HSP)** | **organism** | **accession** | **e-value** | **aa similarity** |
| --- | --- | --- | --- | --- | --- | --- |
| comp306101_c0_seq1 | OalgSRCR2 | macrophage receptor marco | *Mus musculus* | 2OY3 | 7.08E-15 | 68% |
| comp309069_c0_seq1 | OalgSRCR3 | DMBT1-like protein | *Strongylocentrotus purpuratus* | XP_001181916.1 | 4.99E-29 | 63% |
| comp319455_c1_seq1 | OalgSRCR4 | M160/CD163 | *Homo sapiens* | Q9NR16.2 | 1.32E-13 | 72% |
| comp322367_c1_seq1 | OalgSRCR6 | DMBT1-like protein | *Strongylocentrotus purpuratus* | XP_001183040.1 | 1.31E-21 | 68% |
| comp324195_c1_seq2 | OalgSRCR7 | DMBT1-like protein | *Strongylocentrotus purpuratus* | XP_001181916.1 | 7.75E-08 | 66% |
| comp327181_c0_seq1 | OalgSRCR9 | scavenger receptor cysteine-rich type 1 protein M160 | *Callithrix jacchus* | XP_002752336.1 | 3.64E-11 | 77% |
| comp503880_c0_seq1 | OalgSRCR10 | scavenger receptor cysteine-rich protein type 12 | *Strongylocentrotus purpuratus* | XP_001202087.1 | 2.89E-08 | 62% |
| comp740883_c0_seq1 | OalgSRCR11 | SRCR domain, membrane form 2 | *Strongylocentrotus purpuratus* | XP_796328.1 | 1.08E-07 | 73% |
| comp329845_c9_seq3 | OalgSRCR12 | SRCR domain, membrane form 2 | *Geodia cydonium* | CAA75175.1 | 2.66E-22 | 54% |
| comp330058_c4_seq1 | OalgSRCR13 | macrophage receptor marco | *Mus musculus* | 2OY3 | 4.69E-27 | 69% |
| comp331265_c0_seq5 | OalgSRCR15 | scavenger receptor cysteine-rich protein | *Strongylocentrotus purpuratus* | XP_795482.2 | 2.49E-12 | 62% |
| comp332789_c1_seq1 | OalgSRCR17 | scavenger receptor cysteine-rich protein | *Strongylocentrotus purpuratus* | NP_999650.1 | 2.54E-43 | 62% |
| comp334332_c0_seq6 | OalgSRCR18 | Pema-SRCR protein precursor | *Petromyzon marinus* | AAA90990.1 | 1.88E-25 | 67% |
| comp334882_c0_seq1 | OalgSRCR19 | DMBT1-like protein | *Anolis carolinensis* | XP_003228360.1 | 5.22E-19 | 65% |
| comp334995_c0_seq2 | OalgSRCR20 | Pema-SRCR protein precursor | *Petromyzon marinus* | AAA90990.1 | 1.93E-12 | 73% |
| comp334998_c0_seq1 | OalgSRCR21 | M130/CD163 | *Mus musculus* | Q2VLH6.1 | 1.11E-12 | 60% |
| comp334998_c2_seq1 | OalgSRCR22 | scavenger receptor cysteine-rich protein | *Strongylocentrotus purpuratus* | XP_794349.2 | 1.42E-07 | 80% |
| comp333407_c3_seq19 | OalgSRCR25 | SRCR domain, membrane form 2 | *Strongylocentrotus purpuratus* | XP_796328.1 | 2.63E-17 | 63% |

## Additional file Table 12: Host Toll-like receptor sequences

| **transcript ID** | **Sequence Description** | **ncbi accession** | **common name** | **organism** | **BlastHit Description (HSP)** | **e-value** | **aa sim.** |
| --- | --- | --- | --- | --- | --- | --- | --- |
| comp179841_c0_seq1 | variable lymphocyte receptor | BAF43120.1 | arctic lamprey | *Lethenteron japonicum* | variable lymphocyte receptor | 4.36E-12 | 65% |
| comp244649_c0_seq1 | variable lymphocyte receptor | ABO21284.1 | sea lamprey | *Petromyzon marinus* | variable lymphocyte receptor | 1.84E-07 | 64% |
| comp263075_c0_seq1 | variable lymphocyte receptor | ABO21194.1 | sea lamprey | *Petromyzon marinus* | variable lymphocyte receptor | 7.08E-07 | 62% |
| comp288229_c0_seq1 | toll-like receptor | ABK88278.1 | horseshoe crab | *Carcinoscorpius rotundicauda* | toll-like receptor | 5.70E-12 | 65% |
| comp308544_c0_seq1 | toll-like receptor | XP_551799.2 | mosquito | *Anopheles gambiae* | toll-like receptor | 1.36E-10 | 63% |
| comp321901_c0_seq1 | variable lymphocyte receptor | BAF43195.1 | arctic lamprey | *Lethenteron japonicum* | variable lymphocyte receptor | 2.31E-13 | 72% |
| comp321901_c1_seq1 | variable lymphocyte receptor | ABO85960.1 | sea lamprey | *Petromyzon marinus* | variable lymphocyte receptor | 5.25E-10 | 62% |
| comp324324_c1_seq1 | variable lymphocyte receptor | ABA40047.1 | sea lamprey | *Petromyzon marinus* | variable lymphocyte receptor | 5.43E-07 | 49% |
| comp325011_c2_seq1 | variable lymphocyte receptor | ABO21194.1 | sea lamprey | *Petromyzon marinus* | variable lymphocyte receptor | 3.42E-09 | 64% |
| comp329847_c2_seq10 | toll-like receptor | ABK88278.1 | horseshoe crab | *Carcinoscorpius rotundicauda* | toll-like receptor | 5.36E-15 | 42% |
| comp329847_c2_seq12 | leucine rich repeat containing protein 15 | NP_001128529.2 | human | *Homo sapiens* | leucine-rich repeat-cont. protein 15 | 1.43E-45 | 51% |
| comp329847_c2_seq14 | leucine rich repeat containing protein 15 | NP_659551.1 | rat | *Rattus norvegicus* | leucine-rich repeat-cont. protein 15 | 1.83E-45 | 50% |
| comp329847_c2_seq15 | toll-like receptor | ABK88278.1 | horseshoe crab | *Carcinoscorpius rotundicauda* | toll-like receptor | 9.77E-14 | 42% |
| comp329847_c2_seq16 | variable lymphocyte receptor | BAF43202.1 | arctic lamprey | *Lethenteron japonicum* | variable lymphocyte receptor | 1.93E-07 | 45% |
| comp329847_c2_seq17 | variable lymphocyte receptor | BAI66869.1 | inshore hagfish | *Eptatretus burgeri* | variable lymphocyte receptor A | 1.74E-41 | 63% |
| comp329847_c2_seq1 | toll-like receptor | XP_551799.2 | mosquito | *Anopheles gambiae* | toll-like receptor | 1.97E-09 | 69% |
| comp329847_c2_seq3 | toll-like receptor | ABK88278.1 | horseshoe crab | *Carcinoscorpius rotundicauda* | toll-like receptor | 2.18E-23 | 45% |
| comp329847_c2_seq4 | leucine rich repeat containing protein 15 | NP_659551.1 | rat | *Rattus norvegicus* | leucine-rich repeat-cont. protein 15 | 2.05E-45 | 50% |
| comp329847_c2_seq5 | toll-like receptor | ABK88278.1 | horseshoe crab | *Carcinoscorpius rotundicauda* | toll-like receptor | 2.66E-24 | 45% |
| comp329847_c2_seq7 | leucine rich repeat containing protein 15 | NP_659551.1 | rat | *Rattus norvegicus* | leucine-rich repeat-cont. protein 15 | 1.95E-45 | 50% |
| comp329847_c2_seq8 | toll-like receptor | ABK88278.1 | horseshoe crab | *Carcinoscorpius rotundicauda* | toll-like receptor | 3.87E-19 | 44% |
| comp329847_c2_seq9 | leucine rich repeat containing protein 15 | NP_659551.1 | rat | *Rattus norvegicus* | leucine-rich repeat-cont. protein 15 | 2.09E-45 | 50% |
| comp330118_c1_seq11 | variable lymphocyte receptor | XP_001846467.1 | mosquito | *Culex quinquefasciatus* | leucine-rich repeat-cont. protein 15 | 4.19E-07 | 63% |
| comp330118_c1_seq12 | toll protein | ABK58729.1 | Pacific white shrimp | *Litopenaeus vannamei* | toll protein | 2.19E-08 | 42% |
| comp330118_c1_seq13 | toll protein | ABO38434.1 | black tiger shrimp | *Penaeus monodon* | toll receptor | 4.22E-08 | 44% |
| comp330118_c1_seq14 | toll protein | ABO38434.1 | black tiger shrimp | *Penaeus monodon* | toll receptor | 1.22E-07 | 46% |
| comp330118_c1_seq2 | toll protein | ABK58729.1 | Pacific white shrimp | *Litopenaeus vannamei* | toll protein | 2.21E-08 | 42% |
| comp330118_c1_seq5 | variable lymphocyte receptor | ABO15189.1 | sea lamprey | *Petromyzon marinus* | variable lymphocyte receptor | 8.48E-08 | 61% |
| comp330118_c1_seq7 | variable lymphocyte receptor | ABO15189.1 | sea lamprey | *Petromyzon marinus* | variable lymphocyte receptor | 6.34E-08 | 58% |
| comp330178_c1_seq1 | toll-like receptor | AAO53555.1 | goldfish | *Carassius auratus* | toll-like receptor | 7.09E-07 | 57% |
| comp335045_c1_seq6 | toll-like receptor | NP_001133860.1 | salmon | *Salmo salar* | toll-like receptor 13 | 9.38E-07 | 54% |

## Additional file Table 13: Toll immune signaling pathway in *Olavius*

Further components of the toll pathway in *Olavius algarvensis* were identified by tblastx/blastx searches using previously described sequences from annelids (*Capitella teleta*, *Helobdella robusta*, given are trace archive accession numbers) [<davidson2008>] and model organisms (*Drosophila melanogaster*, *Homo sapiens*) as queries against the *Olavius algarvensis* transcriptome assembly; n.a., protein not identified in *C. teleta*/*H. robusta*; ------------ no query; aa, amino acid.

|  | **blast query sequence identifiers (annelids: trace archive sequence IDs)** | | | **respective best blast hits in *Olavius* transcriptome (% aa seq similarity/% seq identity)** | | |
| --- | --- | --- | --- | --- | --- | --- |
| **Protein** | ***Capitella teleta*** | ***Helobdella robusta*** | **uniprot accession** | ***Capitella teleta*** | ***Helobdella robusta*** | **uniprot** |
| ***Drosophila melanogaster*** | | | | | | |
| Cactus | 1109683694 | 1112642406 | ------------ | comp331734_c2 (60/47) | comp329028_c3 (67/44) | ------------ |
| Dorsal | 1027947919 | 1122215071 | ------------ | comp334686_c3 (82/69) | comp334686_c3 (73/53) | ------------ |
| ECSIT | 1085544040 | 1115381906 | ------------ | comp335923_c4 (73/50) | comp335923_c4 (81/66) | ------------ |
| Pelle | 1112699057 | n.a. | ------------ | comp289883_c1 (79/66) | ------------ | ------------ |
| Relish | n.a. | n.a. | Q94527 | ------------ | ------------ | comp330391_c0 (50/34) |
| Spaetzle | n.a. | n.a. | P48607 | ------------ | ------------ | no hit |
| TAK1 | n.a. | n.a. | Q9V3Q6 | ------------ | ------------ | comp329383_c9 (75/57) |
| TRAF1 | 1028363621 | 1112642913 | ------------ | comp334076_c3 (63/46) | comp333584_c10 (74/47) | ------------ |
| Tube | n.a. | n.a. | P22812 | ------------ | ------------ | no hit |
| **Homo sapiens (MyD88-dependent toll signaling)** | | | | | | |
| ECSIT | 1085544040 | 1115381906 | ------------ | comp335923_c4 (72/54) | comp335923_c4 (76/57) | ------------ |
| IKK β | n.a. | n.a. | O14920 | ------------ | ------------ | comp329208_c1 (50/33) |
| IκB ε | 1074119071 | n.a. | ------------ | comp331734_c2 (70/51) | ------------ | ------------ |
| IKK α | n.a. | n.a. | O15111 | ------------ | ------------ | comp307227_c0 (58/34) |
| IRAK1 | n.a. | n.a. | P51617 | ------------ | ------------ | comp334746_c11 (64/47) |
| MEKK | 1026033689 | n.a. | Q13233 | ------------ | ------------ | comp308092_c0 (54/32) |
| MyD88 | 1320293219 | 1343967404 | ------------ | no hit | no hit | ------------ |
| NEMO | n.a. | n.a. | Q9Y6K9 | ------------ | ------------ | no hit |
| NF-κB p105 | n.a. | n.a. | P19838 | ------------ | ------------ | comp330391_c0 (68/53) |
| NF-κB p65 | 1028298985 | n.a. | Q04206 | comp329272_c3 (81/67) | ------------ | comp329272_c3 (66/50) |
| TRAF5 | 1085417814 | 1140751855 | ------------ | comp333584_c10 (77/57) | comp333584_c10 (88/58) | ------------ |
| **Homo sapiens (MyD88-independent toll signaling)** | | | | | | |
| TICAM1 | n.a. | n.a. | Q8IUC6 | ------------ | ------------ | no hit |
| TICAM2 | n.a. | n.a. | Q86XR7 | ------------ | ------------ | no hit |
| TIRAP | n.a. | n.a. | P58753 | ------------ | ------------ | no hit |

## Additional file Table 14: Subcellular localization evidence of host digestive proteins

Table is too large; refer to refer to supplementary file “Supplementary_Table14.xlsx”

#

## Additional file **Figure 1: Transcriptome annotation statistics**

Blast2GO annotation statistics of assembled transcripts; with interproscan: sequences with at least one hit to any of the following databases: ProDom [<corpet1998>], Prints [<attwood1994>], PIR [<wu2002>], Pfam [<finn2008>], Smart [<letunic2004>], TIGRFAM [<haft2003>], PROSITE [<hulo2006>], HAMAP [<pedruzzi2015>], SuperFamily [<wilson2007>], SignalP [<petersen2011>], TMHMM [<krogh2001>], Panther [<mi2005>], Gene3D [<pearl2005>], Phobius [<kaell2004>], Coils [<lupas1991>]; with blast hits: sequences with at least one significant blastx hit to ncbi nr database (e-value cut-off: 1e-6), but no GO mapping or annotation; with mapping: sequences with GO mapping, but no annotation; with annotation: sequences with blast hits and GO mapping, automatically annotated according to blast2go annotation rules [<conesa2005>].

## Additional file Figure 2: Hydrophobicity cluster analysis plots of annelid hemoglobin chains

Two-dimensional hydrophobicity cluster analysis (HCA) plots of selected annelid and *Olavius algarvensis* hemoglobin chains, generated with drawhca [<callebaut1997>]. *Arenicola marina* (GenBank accession numbers: A2c, CAJ32741; B1, CAJ32742; B2, CAI56309), *Ridgeia piscesae* (GenBank accession numbers: A1, ABD72632; A2, ABD72633; B1a, ABD72634; B2, AAP04527), *Riftia pachyptila* (GenBank accession numbers: A1, ABW24412; A2, CAD29155; B1a, CAD29156; B2, CAD29159), *Lamellibrachia satsuma* (GenBank accession numbers: A1, BAN58230; A2, BAN58231; B1, BAN58232; B2, BAN58233), *Lamellibrachia sp*. XB-2003 (GenBank accession numbers: A1, AAP40327; A2, AAP04528; B1, AAP40328; B2, AAP04529), *Oasisia alvinae* (GenBank accession numbers: A2, AAP04531; B2, AAP40329), *Tevnia jerichonana* (GenBank accession number: A2, AAP04530), *Oligobrachia mashikoi* (GenBank accession numbers: A1, Q7M419; A2, Q7M413; B1, Q5KSB7; B2, Q7M418), *Sabella spallanzanii* (GenBank accession numbers: A2, CAC37412; B2a, CAC37410), *Tylorrhynchus heterochaetus* (GenBank accession numbers: A1, P02219; A2, P09966; B2a, P13578), *Lumbricus terrestris* (GenBank accession numbers: A1, P08924; A2, P02218; B1, P11069; B2, P13579), *Lumbricus rubellus* (GenBank accession numbers: A1a, DR009556; A2, BF422675; B1, CAA09958; B2, BF422540), *Olavius algarvensis*, sequences obtained in this study, accession numbers in figure.

## Additional file **Figure 3: Domain structures of proteins with scavenger domains**

Structure of conserved functional domains in *Olavius algarvensis* scavenger receptor cysteine-rich (SRCR) domain containing proteins; Question marks show uncertain sequential arrangement of domains, due to fragmented transcript assembly.

## Additional file **Figure 1: Transcriptome annotation statistics**

Blast2GO annotation statistics of assembled transcripts; with interproscan: sequences with at least one hit to any of the following databases: ProDom [<corpet1998>], Prints [<attwood1994>], PIR [<wu2002>], Pfam [<finn2008>], Smart [<letunic2004>], TIGRFAM [<haft2003>], PROSITE [<hulo2006>], HAMAP [<pedruzzi2015>], SuperFamily [<wilson2007>], SignalP [<petersen2011>], TMHMM [<krogh2001>], Panther [<mi2005>], Gene3D [<pearl2005>], Phobius [<kaell2004>], Coils [<lupas1991>]; with blast hits: sequences with at least one significant blastx hit to ncbi nr database (e-value cut-off: 1e-6), but no GO mapping or annotation; with mapping: sequences with GO mapping, but no annotation; with annotation: sequences with blast hits and GO mapping, automatically annotated according to blast2go annotation rules [<conesa2005>].

**
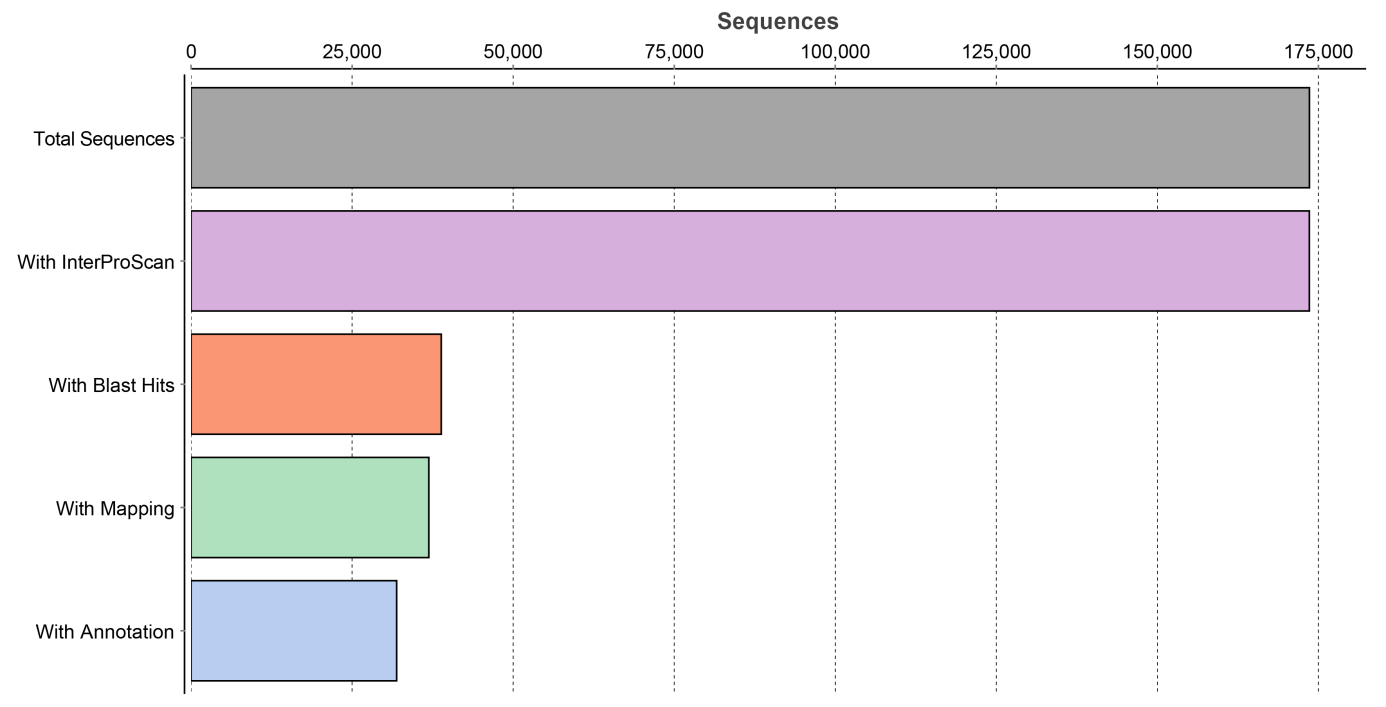
**

## Additional file Figure 2: Hydrophobicity cluster analysis plots of annelid hemoglobin chains

Two-dimensional hydrophobicity cluster analysis (HCA) plots of selected annelid and *Olavius algarvensis* hemoglobin chains, generated with drawhca [<callebaut1997>]. *Arenicola marina* (GenBank accession numbers: A2c, CAJ32741; B1, CAJ32742; B2, CAI56309), *Ridgeia piscesae* (GenBank accession numbers: A1, ABD72632; A2, ABD72633; B1a, ABD72634; B2, AAP04527), *Riftia pachyptila* (GenBank accession numbers: A1, ABW24412; A2, CAD29155; B1a, CAD29156; B2, CAD29159), *Lamellibrachia satsuma* (GenBank accession numbers: A1, BAN58230; A2, BAN58231; B1, BAN58232; B2, BAN58233), *Lamellibrachia sp*. XB-2003 (GenBank accession numbers: A1, AAP40327; A2, AAP04528; B1, AAP40328; B2, AAP04529), *Oasisia alvinae* (GenBank accession numbers: A2, AAP04531; B2, AAP40329), *Tevnia jerichonana* (GenBank accession number: A2, AAP04530), *Oligobrachia mashikoi* (GenBank accession numbers: A1, Q7M419; A2, Q7M413; B1, Q5KSB7; B2, Q7M418), *Sabella spallanzanii* (GenBank accession numbers: A2, CAC37412; B2a, CAC37410), *Tylorrhynchus heterochaetus* (GenBank accession numbers: A1, P02219; A2, P09966; B2a, P13578), *Lumbricus terrestris* (GenBank accession numbers: A1, P08924; A2, P02218; B1, P11069; B2, P13579), *Lumbricus rubellus* (GenBank accession numbers: A1a, DR009556; A2, BF422675; B1, CAA09958; B2, BF422540), *Olavius algarvensis*, sequences obtained in this study, accession numbers in figure.


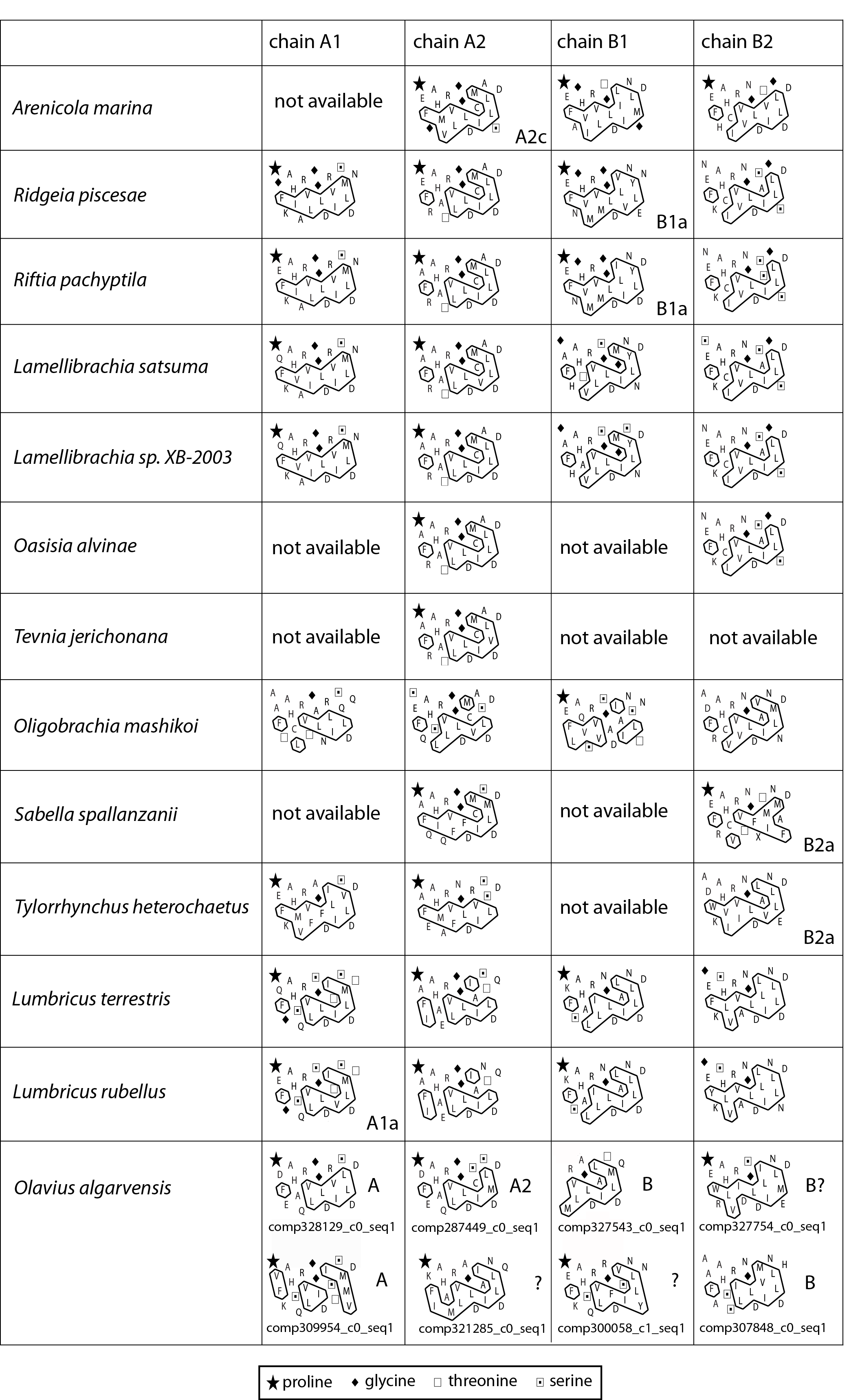


## Additional file **Figure 3: Domain structures of proteins with scavenger domains**

Structure of conserved functional domains in *Olavius algarvensis* scavenger receptor cysteine-rich (SRCR) domain containing proteins; Question marks show uncertain sequential arrangement of domains, due to fragmented transcript assembly.


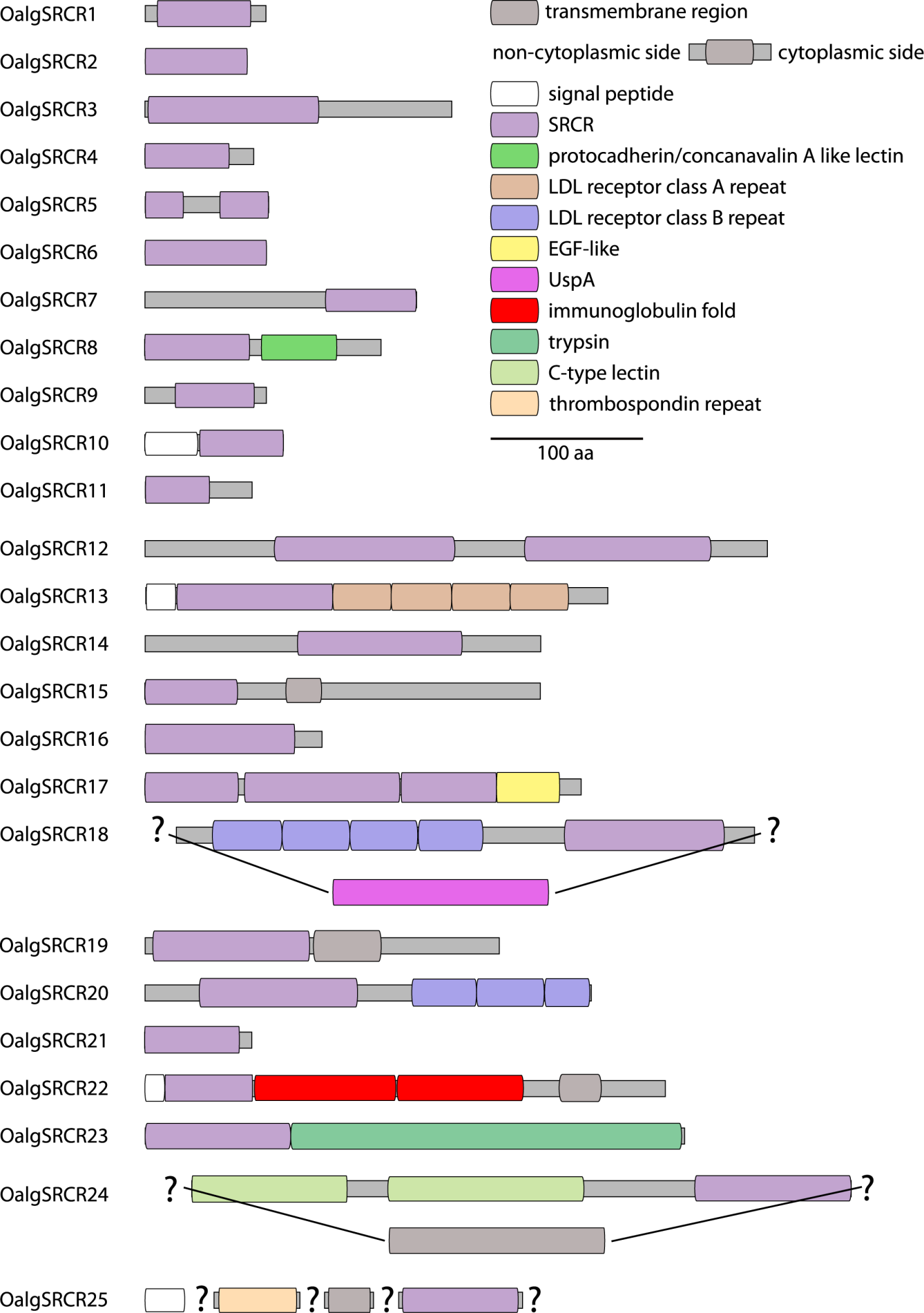


# Supplementary References

[1] Corpet F, Gouzy J, Kahn D. The ProDom database of protein domain families. Nucleic Acids Res. 1998;26:323–326.

[2] Attwood TK, Beck ME, Bleasby AJ, Parry-Smith DJ. PRINTS–a database of protein motif fingerprints. Nucleic Acids Res. 1994;22:3590–3596.

[3] Wu CH, Huang H, Arminski L, Castro-Alvear J, Chen Y, Hu ZZ, Ledley RS, Lewis KC, Mewes HW, Orcutt BC, Suzek BE, Tsugita A, Vinayaka CR, Yeh LSL, Zhang J, Barker WC. The Protein Information Resource: an integrated public resource of functional annotation of proteins. Nucleic Acids Res. 2002;30:35–37.

[4] Finn RD, Tate J, Mistry J, Coggill PC, Sammut SJ, Hotz HR, Ceric G, Forslund K, Eddy SR, Sonnhammer ELL, Bateman A. The Pfam protein families database. Nucleic Acids Res. 2008;36:D281–D288.

[5] Letunic I, Copley RR, Schmidt S, Ciccarelli FD, Doerks T, Schultz J, Ponting CP, Bork P. SMART 4.0: towards genomic data integration. Nucleic Acids Res. 2004;32:D142–D144.

[6] Haft DH, Selengut JD, White O. The TIGRFAMs database of protein families. Nucleic Acids Res. 2003;31:371–373.

[7] Hulo N, Bairoch A, Bulliard V, Cerutti L, De Castro E, Langendijk-Genevaux PS, Pagni M, Sigrist CJA. The PROSITE database. Nucleic Acids Res. 2006;34:D227–D230.

[8] Wilson D, Madera M, Vogel C, Chothia C, Gough J. The SUPERFAMILY database in 2007: families and functions. Nucleic Acids Res. 2007;35:D308–D313.

[9] Petersen TN, Brunak S, von Heijne G, Nielsen H. SignalP 4.0: discriminating signal peptides from transmembrane regions. Nat Methods. 2011;8:785–786.

[10] Krogh A, Larsson B, von Heijne G, Sonnhammer EL. Predicting transmembrane protein topology with a hidden Markov model: application to complete genomes. J Mol Biol. 2001;305:567–580.

[11] Mi H, Lazareva-Ulitsky B, Loo R, Kejariwal A, Vandergriff J, Rabkin S, Guo N, Muruganujan A, Doremieux O, Campbell MJ, Kitano H, Thomas PD. The PANTHER database of protein families, subfamilies, functions and pathways. Nucleic Acids Res. 2005;33:D284–D288.

[12] Pearl F, Todd A, Sillitoe I, Dibley M, Redfern O, Lewis T, Bennett C, Marsden R, Grant A, Lee D, Akpor A, Maibaum M, Harrison A, Dallman T, Reeves G, Diboun I, Addou S, Lise S, Johnston C, Sillero A, Thornton J, Orengo C. The CATH Domain Structure Database and related resources Gene3D and DHS provide comprehensive domain family information for genome analysis. Nucleic Acids Res. 2005;33:D247–D251.

[13] Käll L, Krogh A, Sonnhammer ELL. A combined transmembrane topology and signal peptide prediction method. J Mol Biol. 2004;338:1027–1036.

[14] Lupas A, Van Dyke M, Stock J. Predicting coiled coils from protein sequences. Science. 1991;252:1162–1164.

[15] Kleiner M, Wentrup C, Lott C, Teeling H, Wetzel S, Young J, Chang YJ, Shah M, VerBerkmoes NC, Zarzycki J, Fuchs G, Markert S, Hempel K, Voigt B, Becher D, Liebeke M, Lalk M, Albrecht D, Hecker M, Schweder T, Dubilier, N. Metaproteomics of a gutless marine worm and its symbiotic microbial community reveal unusual pathways for carbon and energy use. Proc Nat Acad Sci U S A. 2012;109:E1173–E1182.

[16] Woyke T, Teeling H, Ivanova NN, Huntemann M, Richter M, Gloeckner FO, Boffelli D, Anderson IJ, Barry KW, Shapiro HJ, Szeto E, Kyrpides NC, Mussmann M, Amann R, Bergin C, Ruehland C, Rubin EM, Dubilier, N. Symbiosis insights through metagenomic analysis of a microbial consortium. Nature. 2006;443:950–955.

[17] Pĕtra PH, Hermodson MA, Walsh KA, Neurath H. Characterization of bovine carboxypeptidase A (Allan). Biochemistry. 1971;10:4023–4025.

[18] Catasús L, Villegas V, Pascual R, Avilés FX, Wicker-Planquart C, Puigserver A. cDNA cloning and sequence analysis of human pancreatic procarboxypeptidase A1. Biochem J. 1992;287:299–303.

[19] Quinto C, Quiroga M, Swain WF, Nikovits W Jr, Standring DN, Pictet RL, Valenzuela P, Rutter WJ. Rat preprocarboxypeptidase A: cDNA sequence and preliminary characterization of the gene. Proc Natl Acad Sci U S A. 1982;79:31–35.

[20] Catasús L, Vendrell J, Avilés FX, Carreira S, Puigserver A, Billeter M. The sequence and conformation of human pancreatic procarboxypeptidase A2. cDNA cloning, sequence analysis, and three-dimensional model. J Biol Chem. 1995;270:6651–6657.

[21] Gardell SJ, Craik CS, Clauser E, Goldsmith EJ, Stewart CB, Graf M, Rutter WJ. A novel rat carboxypeptidase, CPA2: characterization, molecular cloning, and evolutionary implications on substrate specificity in the carboxypeptidase gene family. J Biol Chem. 1988;263:17828–17836.

[22] Edwards MJ, Moskalyk LA, Donelly-Doman M, Vlaskova M, Noriega FG, Walker VK, Jacobs-Lorena M. Characterization of a carboxypeptidase A gene from the mosquito, *Aedes aegypti*. Insect Mol Biol. 2000;9:33–38.

[23] Bown DP, Wilkinson HS, Gatehouse JA. Midgut carboxypeptidase from *Helicoverpa armigera* (Lepidoptera: Noctuidae) larvae: enzyme characterisation, cDNA cloning and expression. Insect Biochem Mol Biol. 1998;28:739–749.

[24] Jochim RC, Teixeira CR, Laughinghouse A, Mu J, Oliveira F, Gomes RB, Elnaiem DE, Valenzuela JG. The midgut transcriptome of *Lutzomyia longipalpis*: comparative analysis of cDNA libraries from sugar-fed, blood-fed, post-digested and *Leishmania infantum chagasi*-infected sand flies. BMC Genomics. 2008;9:15.

[25] Ribeiro JMC, Genta FA, Sorgine MHF, Logullo R, Mesquita RD, Paiva-Silva GO, Majerowicz D, Medeiros M, Koerich L, Terra WR, Ferreira C, Pimentel AC, Bisch PM, Leite DC, Diniz MMP, da S G V Junior JL, Da Silva ML, Araujo RN, Gandara ACP, Brosson S, Salmon D, Bousbata S, González-Caballero N, Silber AM, Alves-Bezerra M, Gondim KC, Silva-Neto MAC, Atella GC, Araujo H, Dias FA, Polycarpo C, Vionette-Amaral RJ, Fampa P, Melo ACA, Tanaka AS, Balczun C, Oliveira JHM, Gonçalves RLS, Lazoski C, Rivera-Pomar R, Diambra L, Schaub GA, Garcia ES, Azambuja P, Braz GRC, Oliveira PL. An insight into the transcriptome of the digestive tract of the bloodsucking bug, *Rhodnius prolixus*. PLoS Negl Trop Dis. 2014;8:e2594.

[26] Prabhakar S, Chen MS, Elpidina EN, Vinokurov KS, Smith CM, Marshall J, Oppert B. Sequence analysis and molecular characterization of larval midgut cDNA transcripts encoding peptidases from the yellow mealworm, *Tenebrio molitor* L. Insect Mol Biol. 2007;16:455–468.

[27] Wang P, Li G, Kain W. Characterization and cDNA cloning of midgut carboxypeptidases from *Trichoplusia ni*. Insect Biochem Mol Biol. 2004;34:831–843.

[28] Lee MS, Cho SJ, Tak ES, Lee JA, Cho HJ, Park BJ, Shin C, Kim DK, Park SC. Transcriptome analysis in the midgut of the earthworm (*Eisenia andrei*) using expressed sequence tags. Biochem Biophys Res Commun. 2005;328:1196–1204.

[29] Hartley BS. Amino-acid sequence of the bovine chymotrypsinogen-A. Nature. 1964;201:1284–1287.

[30] Asgeirsson B, Bjarnason JB. Structural and kinetic properties of chymotrypsin from Atlantic cod (*Gadus morhua*). Comparison with bovine chymotrypsin. Comp Biochem Physiol B. 1991;99:327–335.

[31] Pinsky SD, LaForge KS, Luc V, Scheele G. Identification of cDNA clones encoding secretory isoenzyme forms: sequence determination of canine pancreatic prechymotrypsinogen 2 mRNA. Proc Natl Acad Sci U S A. 1983;80:7486–7490.

[32] Spilliaert R, Gudmundsdóttir A. Molecular cloning of the Atlantic cod chymotrypsinogen B. Microb Comp Genomics. 2000;5:41–50.

[33] Tomita N, Izumoto Y, Horii A, Doi S, Yokouchi H, Ogawa M, Mori T, Matsubara K. Molecular cloning and nucleotide sequence of human pancreatic prechymotrypsinogen cDNA. Biochem Biophys Res Commun. 1989;158:569–575.

[34] Bell GI, Quinto C, Quiroga M, Valenzuela P, Craik CS, Rutter WJ. Isolation and sequence of a rat chymotrypsin B gene. J Biol Chem. 1984;259:14265–14270.

[35] Mazumdar-Leighton S, Broadway RM. Identification of six chymotrypsin cDNAs from larval midguts of *Helicoverpa zea* and *Agrotis ipsilon* feeding on the soybean (Kunitz) trypsin inhibitor. Insect Biochem Mol Biol. 2001;31:633–644.

[36] Degnan BM, Groppe JC, Morse DE. Chymotrypsin mRNA expression in digestive gland amoebocytes: cell specification occurs prior to metamorphosis and gut morphogenesis in the gastropod, *Haliotis rufescens*. Rouxs Arch Dev Biol. 1995;205:97–101.

[37] Chougule NP, Giri AP, Sainani MN, Gupta VS. Gene expression patterns of *Helicoverpa armigera* gut proteases. Insect Biochem Mol Biol. 2005;35:355–367.

[38] Zhu YC, Guo Z, Abel C. Cloning eleven midgut trypsin cDNAs and evaluating the interaction of proteinase inhibitors with Cry1Ac against the tobacco budworm, *Heliothis virescens* (F.) (Lepidoptera: Noctuidae). J Invertebr Pathol. 2012;111:111–120.

[39] Zhu YC, Liu X, Maddur AA, Oppert B, Chen MS. Cloning and characterization of chymotrypsin- and trypsin-like cDNAs from the gut of the Hessian fly [*Mayetiola destructor* (Say)]. Insect Biochem Mol Biol. 2005;35:23–32.

[40] Balti R, Bougherra F, Bougatef A, Hayet BK, Nedjar-Arroume N, Dhulster P, Guillochon D, Nasri M. Chymotrypsin from the hepatopancreas of cuttlefish (*Sepia officinalis*) with high activity in the hydrolysis of long chain peptide substrates: Purification and biochemical characterisation. Food Chem. 2012;130:475–484.

[41] Elpidina EN, Tsybina TA, Dunaevsky YE, Belozersky MA, Zhuzhikov DP, Oppert B. A chymotrypsin-like proteinase from the midgut of *Tenebrio molitor* larvae. Biochimie. 2005;87:771–779.

[42] Reseland JE, Larsen F, Solheim J, Eriksen JA, Hanssen LE, Prydz H. A novel human chymotrypsin-like digestive enzyme. J Biol Chem. 1997;272:8099–8104.

[43] Largman C, Brodrick JW, Geokas MC. Purification and characterization of two human pancreatic elastases. Biochemistry. 1976;15:2491–2500.

[44] Largman C. Isolation and characterization of rat pancreatic elastase. Biochemistry. 1983;22:3763–3770.

[45] Gertler A, Birk Y. Isolation and characterization of porcine proelastase. Eur J Biochem. 1970;12:170–176.

[46] Qiu R, Liu X, Hu YH, Sun BG. Expression characterization and activity analysis of a cathepsin B from Pacific abalone *Haliotis discus hannai*. Fish Shellfish Immunol. 2013;34:1376–1382.

[47] Stephens A, Rojo L, Araujo-Bernal S, Garcia-Carreño F, Muhlia-Almazan A. Cathepsin B from the white shrimp *Litopenaeus vannamei*: cDNA sequence analysis, tissues-specific expression and biological activity. Comp Biochem Physiol B Biochem Mol Biol. 2012;161:32–40.

[48] Wang X, Liu B, Wang G, Tang B, Xiang J. Molecular cloning and functional analysis of cathepsin B in nutrient metabolism during larval development in *Meretrix meretrix*. Aquaculture. 2008;282:41–46.

[49] Ranjit N, Zhan B, Stenzel DJ, Mulvenna J, Fujiwara R, Hotez PJ, Loukas A. A family of cathepsin B cysteine proteases expressed in the gut of the human hookworm, *Necator americanus*. Mol Biochem Parasitol. 2008;160:90–99.

[50] Pinlaor P, Kaewpitoon N, Laha T, Sripa B, Kaewkes S, Morales ME, Mann VH, Parriott SK, Suttiprapa S, Robinson MW, To J, Dalton JP, Loukas A, Brindley PJ. Cathepsin F cysteine protease of the human liver fluke, *Opisthorchis viverrini*. PLoS Negl Trop Dis. 2009;3:e398.

[51] Aoki H, Ahsan MN, Watabe S. Molecular cloning and characterization of cathepsin B from the hepatopancreas of northern shrimp *Pandalus borealis*. Comp Biochem Physiol B Biochem Mol Biol. 2003;134:681–694.

[52] Fuzita FJ, Pinkse MWH, Patane JSL, Juliano MA, Verhaert PDEM, Lopes AR. Biochemical, transcriptomic and proteomic analyses of digestion in the scorpion *Tityus serrulatus*: insights into function and evolution of digestion in an ancient arthropod. PLoS One. 2015;10:e0123841.

[53] Kang JM, Bahk YY, Cho PY, Hong SJ, Kim TS, Sohn WM, Na BK. A family of cathepsin F cysteine proteases of *Clonorchis sinensis* is the major secreted proteins that are expressed in the intestine of the parasite. Mol Biochem Parasitol. 2010;170:7–16.

[54] Wang B, Shi GP, Yao PM, Li Z, Chapman HA, Brömme D. Human cathepsin F. Molecular cloning, functional expression, tissue localization, and enzymatic characterization. J Biol Chem. 1998;273:32000–32008.

[55] Deussing J, Tisljar K, Papazoglou A, Peters C. Mouse cathepsin F: cDNA cloning, genomic organization and chromosomal assignment of the gene. Gene. 2000;251:165–173.

[56] Ahn SJ, Kim NY, Seo JS, Je JE, Sung JH, Lee SH, Kim MS, Kim JK, Chung JK, Lee HH. Molecular cloning, mRNA expression and enzymatic characterization of cathepsin F from olive flounder (*Paralichthys olivaceus*). Comp Biochem Physiol B Biochem Mol Biol. 2009;154:211–220.

[57] Hu X, Hu X, Hu B, Wen C, Xie Y, Wu D, Tao Z, Li A, Gao Q. Molecular cloning and characterization of cathepsin L from freshwater mussel, *Cristaria plicata*. Fish Shellfish Immunol. 2014;40:446–454.

[58] Matsumoto I, Watanabe H, Abe K, Arai S, Emori Y. A putative digestive cysteine proteinase from *Drosophila melanogaster* is predominantly expressed in the embryonic and larval midgut. Eur J Biochem. 1995;227:582–587.

[59] Li WW, Jin XK, He L, Jiang H, Gong YN, Xie YN, Wang Q. Molecular cloning, characterization, expression and activity analysis of cathepsin L in Chinese mitten crab, *Eriocheir sinensis*. Fish Shellfish Immunol. 2010;29:1010–1018.

[60] Gal S, Gottesman MM. Isolation and sequence of a cDNA for human pro-(cathepsin L). Biochem J. 1988;253:303–306.

[61] Hu KJ, Leung PC. Food digestion by cathepsin L and digestion-related rapid cell differentiation in shrimp hepatopancreas. Comp Biochem Physiol B Biochem Mol Biol. 2007;146:69–80.

[62] Portnoy DA, Erickson AH, Kochan J, Ravetch JV, Unkeless JC. Cloning and characterization of a mouse cysteine proteinase. J Biol Chem. 1986;261:14697–14703.

[63] Ma J, Zhang D, Jiang J, Cui S, Pu H, Jiang S. Molecular characterization and expression analysis of cathepsin L1 cysteine protease from pearl oyster *Pinctada fucata*. Fish Shellfish Immunol. 2010;29:501–507.

[64] Ishidoh K, Towatari T, Imajoh S, Kawasaki H, Kominami E, Katunuma N, Suzuki K. Molecular cloning and sequencing of cDNA for rat cathepsin L. FEBS Lett. 1987;223:69–73.

[65] Cristofoletti PT, Ribeiro AF, Terra WR. The cathepsin L-like proteinases from the midgut of *Tenebrio molitor* larvae: sequence, properties, immunocytochemical localization and function. Insect Biochem Mol Biol. 2005;35:883–901.

[66] Grabherr MG, Haas BJ, Yassour M, Levin JZ, Thompson DA, Amit I, Adiconis X, Fan L, Raychowdhury R, Zeng Q, Chen Z. Trinity: reconstructing a full-length transcriptome without a genome from RNA-Seq data. Nature Biotechnol. 2011;29:644.

[67] Sanchez S, Hourdez S, Lallier FH. Identification of proteins involved in the functioning of *Riftia pachyptila* symbiosis by Subtractive Suppression Hybridization. BMC Genomics. 2007;8:1.

[68] Davidson CR, Best NM, Francis JW, Cooper EL, Wood TC. Toll-like receptor genes (TLRs) from *Capitella capitata* and *Helobdella robusta* (Annelida). Dev Comp Immunol. 2008;32:608–612.

[69] Pedruzzi I, Rivoire C, Auchincloss AH, Coudert E, Keller G, de Castro E, Baratin D, Cuche BA, Bougueleret L, Poux S, Redaschi N, Xenarios I, Bridge A. HAMAP in 2015: updates to the protein family classification and annotation system. Nucleic Acids Res. 2015;43:D1064–D1070.

[70] Conesa A, Götz S, Garcá-Gómez JM, Terol J, Talón M, Robles M. Blast2GO: a universal tool for annotation, visualization and analysis in functional genomics research. Bioinformatics. .2005;21:3674–3676

[71] Callebaut I, Labesse G, Durand P, Poupon A, Canard L, Chomilier J, Henrissat B, Mornon J. Deciphering protein sequence information through hydrophobic cluster analysis (HCA): current status and perspectives. Cell Mol Life Sci. 1997;53:621–645.
